# Exploring the Influence of Toasting Levels, Grain Sizes, and Their Combination on the Volatile Profile of Tempranillo Red Wines Aged in *Quercus petraea* Barrels

**DOI:** 10.3390/molecules30061293

**Published:** 2025-03-13

**Authors:** Mikel Landín Ross-Magahy, Leticia Martínez-Lapuente, Belén Ayestarán, Zenaida Guadalupe

**Affiliations:** Institute of Vine and Wine Sciences, ICVV (University of La Rioja, Government of La Rioja and CSIC), Finca La Grajera, 26007 Logroño, Spain; mikel.landin@unirioja.es (M.L.R.-M.); belen.ayestaran@unirioja.es (B.A.)

**Keywords:** French oak, barrel ageing, aroma compounds, wood properties

## Abstract

The ageing of wine in oak barrels enhances its sensory properties, with the toasting level and grain size of the wood significantly influencing the volatile composition of the wine. This study evaluated the impact of three toasting levels (light toasting, medium toasting, and medium long toasting) and two grain sizes (standard grain and extra fine grain) on the volatile profile of Tempranillo red wines aged in *Quercus petraea* barrels over 12 and 18 months. Gas chromatography–mass spectrometry was employed to quantify wine volatile compounds. The results revealed that lighter toasting combined with standard grain barrels enhanced the wine volatile concentration during shorter maturation periods, while medium long toasting with extra fine grain barrels was more effective for longer ageing periods. Toasting level was found to have a stronger influence on the wine volatile composition than grain size. These findings underscore the importance of tailoring barrel specifications to the desired maturation durations and sensory outcomes. Future studies exploring other grape varieties, wood origins, and longer ageing periods could further refine these insights and enhance winemaking practices.

## 1. Introduction

The ageing of wine in oak barrels is a widespread practice that enhances its colour, stability, and aromatic profile. This process contributes to a more complex aroma due to the extraction of volatile compounds from the wood. The volatile compounds released from oak wood into wine can be present naturally in the oak or result from transformations during the toasting process [1]. The main volatile substances released by oak wood include furans, phenolic aldehydes and ketones, volatile phenols, and β-methyl-γ-octalactones [2,3,4], which can contribute to oak aroma with toast, caramel, wood, vanilla, cream, smoky, and spicy notes, amongst others [2].

Barrel-making, and particularly the toasting step, plays a crucial role in shaping the composition and sensory characteristics of barrel-matured wine. During barrel assembly, staves are bent and toasted, a process that alters the wood’s physical and chemical properties, significantly influencing the volatile composition [5,6] and sensory profile of the wines [2,6]. Toasting is classified as light, medium, or heavy depending on the length of the *bousinage* [2]. The duration of toasting affects the wood’s chemical composition in distinct ways, altering the substances released into the wine during maturation and consequently influencing its sensory profile. Toasting involves the thermal degradation of wood macromolecules such as cellulose, hemicelluloses, and lignin, which leads to the formation of new volatile compounds. Several studies have reported the influence of toasting level on volatile substances [7,8,9,10,11]. In fact, the toasting process can increase the concentration of certain compounds [12], such as volatile phenols, phenolic aldehydes, and phenolic alcohols [13], while reducing others, such as *cis* and *trans* oak lactones, as toasting temperature rises [11]. Specifically, it was observed that toasting levels determine the release of key compounds such as vanillin, furfural, guaiacol, and syringol, which are more abundant in medium and heavily toasted barrels compared to lightly toasted ones [10]. The impact of toasting also extends to the release of wood-derived non-volatile compounds during barrel ageing, which further enhances the sensory profile of wine. These compounds contribute to improved balance, reduced astringency, and stabilisation of red wine colour through hydrolysable ellagitannins, particularly in lightly toasted or untoasted wood [14]. Heavily toasted barrels, however, release lower levels of these tannins while imparting distinctive smoky and spicy notes [15].

Grain type is another crucial factor influencing barrel quality and is a key selection criterion for cooperages. Grain size reflects the tree’s annual growth rings [16], which depend on the botanical and geographical origin of the wood [5,17]. Normally, oak is classified as fine grain oak when there are five or more growth rings per cm, as medium-grain oak when there are 3–4 growth rings per cm, and as coarse-grain oak when there are two or fewer growth rings per cm [16]. French oak classification often links grain size to forest origin: Limousin oak is wide-grained, Vosges oak has average grain, and Allier oak is very tight-grained [18]. However, grain size varies significantly among trees within the same forest [2]. For these reasons, some cooperages classify their wood based on the actual grain size rather than its geographical origin [2]. Some studies have shown an existing relationship between grain and wood quality, reporting that larger grain woods were richer in ellagitannins and poorer in aromatic compounds such as eugenol and the β-methyl-γ-octalactone isomers [17]. It was observed that wood grain type affects the concentration of the *cis* isomer of oak lactone, with significantly lower levels in wines aged in extra fine grain barrels compared to those aged in fine grain barrels [11]. However, other authors found that the cooperage toasting process eliminated any differences in the volatile composition of wood that was previously classified by grain size [19].

Additionally, grain size plays a role in the rate of entry or the oxygen transmission rate (OTR) because more oxygen enters when the grain is smaller [20]. In this respect, it was described that red wines aged in oak barrels with different OTRs develop distinct profiles, as wines matured in low OTR barrels were characterised by 4-ethylguaiacol, eugenol, 4-vinilguaiacol, guaiacol, and 4-methylguaiacol while high OTR wines were described by *trans* and *cis*-β-methyl-γ-octalactone [21]. However, it is worth noting that a recent work suggests that the association between wood grain with the OTR is not statically significant, despite being important [22,23]. In fact, weak correlations were observed between OTR and factors such as density or grain, both of which are key parameters in wood classification [22,23].

Prior research has explored the impact of different origins [24,25,26], maturation times [7,26,27], and toasting levels [7,8,9,10,11] on the wine’s volatile composition. However, there has been limited investigation into the influence of grain size on the volatile composition of red wine aged in oak barrels and practically no studies have evaluated a combination of both [11]. To our knowledge, this is the first time these two factors have been studied for Tempranillo red wine matured in *Quercus petraea* barrels. Therefore, the aim of this study was to investigate the impact of varying degrees of barrel toasting (light toasting—LT; medium toasting—MT; and medium long toasting—MLT) and grain type (standard grain—SG and extra fine grain—EG) on the volatile composition of Tempranillo red wines aged in *Quercus petraea* barrels. In order to do so, 36 new *Quercus petraea* barrels were used with different toasting levels and grain types. Wine was aged in these barrels for over 12 and 18 months at which point samples were taken to analyse their aromas.

## 2. Results and Discussion

### 2.1. The Percentage of Attributable Variance (%) of the Independent Effect of Toasting Level and Grain Size of the Barrel, and the Interaction of Both (Toasting Level × Grain Size)

Table 1 shows the percentage of variance attributable to toasting level, grain size of the barrel, and their interaction on the wine volatile composition at 12 and 18 months of barrel ageing.

The wine volatile compounds analysed can be grouped into non-oak-related volatiles and oak-related volatiles. The non-oak-related volatiles were formed by the following volatile families: C6 alcohols, higher alcohols, C13-norisprenoids, terpenes, ethyl esters and acetates, fatty acids, γ-lactones, carbonyl compounds, and others. In the case of oak-related volatiles, the families that form them include furanic compounds, lactones, volatile phenols, and phenolic aldehydes.

The toasting level effect (% of variance obtained by the MANOVA analysis) wielded a more substantial influence on the attributable variance compared to the grain size on the volatile profile of aged Tempranillo wine at both the 12- and 18-month maturation periods. The toasting level effect presented no clear patterns of influence whether the volatile compounds were oak or non-oak-related. The percentage of the variance of the toasting level effect was the highest in carbonyl compounds (81.62%) at 12 months of maturation, while volatile phenols (76.88%) were the main compounds affected by toasting level at 18 months of ageing. The percentage of the variance of the grain size effect was the highest in furanic compounds at both 12 and 18 months of maturation (76.78% and 38.46%, respectively).

Specifically, after 12 months, the toasting level exhibited a greater attributable variance across all volatile families except for C13-norisoprenoids and furanic compounds, where grain size demonstrated a higher percentage. Similarly, after 18 months of ageing, a greater effect of toasting compared to grain size was observed for most compounds. However, terpenes did not show statistical significance for the toasting level, carbonyl compounds exhibited a higher variance attributable to the interaction between toasting and grain size, and furanic compounds showed a higher variance attributable to the independent effect of grain size.

It is worth noting that the combined effect of both parameters became more pronounced from the 12- to the 18-month maturation period in 9 out of the 13 volatile families studied. The exceptions were the C13-norisoprenoids, terpenes, lactones, and volatile phenols. Significantly, the interaction of these parameters exerted a notable impact, with percentages exceeding 25% for the C6 alcohols, ethyl esters and acetates, fatty acids, carbonyl compounds, and vanillin. This increase could be explained by the increase in the independent effect of grain size, causing in turn, the increase in the combined effect.

From the 12 to 18 month ageing periods, some changes were observed in the percentage of attributable variance. The toasting percentage increased for all volatile families except for vanillin due to a reduction in the attributable variance for the vanilla content. The impact of grain size was manifested in an increase in the percentage of variance attributable for lactones and phenolic aldehydes, more specifically *cis*-whiskey-lactone and vanillin, which provide woody, coconut, and vanilla aromas, respectively. In contrast, a reduction was noted for furanic compounds associated with bitter almonds, spice, and hay aromas, as well as for volatile phenols characterised by spices, clove, and curry notes.

The toasting level of the barrels had a greater influence than grain size on the volatile profile of Tempranillo wine across most volatile families at both 12 and 18 months of maturation. The grain size accounted for a higher attributable variance in furanic compounds at both ageing periods, in C13-norisoprenoids at 12 months, and in carbonyl compounds at 18 months. The interaction between toasting level and grain size became increasingly pronounced over time, significantly impacting key volatile families, including C6 alcohols, higher alcohols, ethyl esters and acetates, fatty acids, γ-lactones, carbonyl compounds, others, furanic compounds, and vanillin.

### 2.2. Toasting Effect on Volatile Profile of Tempranillo Red Wine Aged in Quercus petraea Barrels

Table 2 shows the Tempranillo red wine concentration (µg 4-nonanol/L) and the odour active value (OAV) for each volatile compound at 12 and 18 months of barrel ageing according to the toasting level applied [light toasting (LT), medium toasting (MT), and medium long toasting (MLT)].

A notable difference was observed in both the toasting level and the ageing period. In fact, wines aged in LT barrels at 12 months and wines aged in MLT barrels at 18 showed the highest concentrations for most wine volatiles.

Wines aged 12 months in LT barrels at 12 showed higher concentrations for all volatile families compared to those aged in MT or MLT barrels except for carbonyl compounds, furanic compounds, and lactones. Although [12] found that furanic compounds increased during toasting in oak woods, no significant differences were observed in the content of carbonyl and furanic compounds, and MLT and LT barrels exhibited higher concentrations of lactones compared to MT barrels.

The higher concentration of volatile compounds in wines aged in LT barrels was particularly evident in C13-norisoprenoids and ‘other’ compounds, which were 40% and 40–64% higher, respectively, compared to those found in wines aged in MT and MLT barrels. Specifically, wines aged in LT barrels for 12 months showed concentrations of β-ionone and β-damascenone over 73% and 32% higher, respectively, than those obtained in wines aged in MT and MLT barrels for 12 months. Similarly, C6 alcohols, higher alcohols, terpenes, ethyl esters and acetates, fatty acids, and γ-lactones showed markedly higher concentrations in wines in LT than MT and MLT barrels, with percentage increases ranging from 21 to 34%. Among oak-related compounds, lactones, volatile phenols, and vanillin displayed concentrations 27% higher in wines in LT barrels compared to MT and MLT barrels. Lactones are heat-sensitive compounds that can be lost by volatilization when the oak wood is subjected to high temperatures or even charring [1,28], which could explain why their concentrations were lower in the wines of matured LT barrels. *Cis* oak lactone displayed a higher content in light toasted barrels compared to higher levels of toasting, which is in agreement with previous research [1,7,8,11,29], and with those describing higher concentrations of lactones are found in wines aged in untoasted oak, as these compounds can be found naturally in oak wood [1,30,31].

After 12 months of ageing, 24 out of 69 quantified volatile compounds (35% of total compounds) were found at average concentrations higher than their corresponding odour thresholds (OAV > 1) in wines aged in LT barrels. Wines aged in MT and MLT barrels showed 22 out of 69 quantified volatile compounds (32% of total compounds) and 24 out of 69 quantified volatile compounds (35% of total compounds), respectively, with concentrations higher than their corresponding odour thresholds (OAV > 1).

Among non-wood-related volatiles, esters compounds, such as ethyl octanoate, ethyl hexanoate, and methyl salicylate, were the most prominent contributors to the fruity and peppermint aroma profile. Ethyl octanoate showed OAVs of 93.79, 73.13, and 61.28 in wines aged in LT, MT, and MLT barrels, respectively, while ethyl hexanoate reached OAVs of 48.38, 29.82, and 32.79, respectively. Lastly, methyl salicylate reached OAVs of 44.18, 25.90, and 22.42 in wines matured in LT, MT, and MLT barrels, respectively. β-damascenone stood out among C13 norisoprenoids for its highest OAV, showing values of 67.42 in wines aged in LT barrels versus 41.59 and 45.73 in wines matured in MT and MLT barrels, respectively. The significantly higher OAVs in LT-aged wines suggested that higher toasting levels degraded non-oak derived volatiles, as seen in previous research [32]. Among the higher alcohols, 2-phenylethanol, characterised by its rose-like and floral aromas, showed OAVs of 8.22 in LT barrels versus 5.69 and 6.23 in MT and MLT barrels, respectively. Its significantly increased concentration in LT-aged wines likely results from a lesser degradation caused by its lower toasting degree.

Among the wood-related volatiles and regardless of the degree of toasting, eugenol and guaiacol stood out due to their high OAV in the volatile phenol family. Eugenol, with OAVs of 15.83, 7.86, and 12.82 in wines aged in LT, MT, and MLT barrels, respectively, is a key contributor to spicy and clove-like aromas. Previous research [9] supports this finding, reporting higher eugenol concentrations in wines treated with lightly toasted chips, which correlates with the elevated levels observed in LT barrels at 12 months. Similarly, guaiacol, which provides smoky and toasty notes, exhibited the highest OAV of 2.66 in wines aged in LT barrels compared to 1.88 and 1.78 in wines matured in MT and MLT barrels. Phenolic aldehydes such as vanillin, responsible for sweet and vanilla-like notes, also showed higher OAVs in LT barrels (1.39) compared to MT and MLT barrels (0.99 and 1.00). Vanillin is primarily synthesised within wood during the toasting process, and its concentration in the wood is indicative of the intensity of the toasting applied [29]. As seen in Table 2, the vanillin concentration and OAV was higher in wines aged in LT barrels, suggesting that lower toasting temperatures improves vanillin extraction [29,33]. In this sense, several authors reported that the high toasting temperature degrades vanillin [34,35], and that this volatile compound increases in light and medium toasting but decreases in heavy toasting [36].

Lastly, lactones like *cis*-whiskey-lactone exhibited a higher OAV in wines aged in LT barrels compared to MT and MLT barrels. Vanillin and *cis*-whiskey-lactone, amongst other volatile compounds (furfural and 5-methyl-furfural), are described as very potent contributors to barrel toasting aromas. Their role is described in the same way as fruity descriptors as they enhance the oaky flavour and act as markers and/or precursors for potent odorants perceived as an oak barrel aroma [4].

After 18 months of ageing, wines aged in MLT barrels showed the highest concentrations of all families except for vanillin where wines aged in LT and MLT barrels showed higher amounts (43–52%) than those matured in MT barrels. Specifically, wines aged in MLT barrels showed significantly higher concentrations than LT and MT barrels in C6-alcohols (23–29%), higher alcohols (33–48%), C13-norisoprenoids (34–36%), terpenes (6–18%), ethyl esters and acetates (33–47%), fatty acids (31–45%), γ-lactones (40–53%), carbonyl compounds (34–44%), ‘others’ (24–38%), furanic compounds (25–43%), lactones (39–48%), and volatile phenols (30–53%).

At 18 months of ageing, 24 out of 69 volatile compounds (35% of total compounds) were found at average concentrations higher than their corresponding odour thresholds (OAV > 1) in wines aged in LT barrels. For wines matured in MT barrels, 22 out of 69 (32% of total compounds) were in concentrations higher than their corresponding odour thresholds (OAV > 1), and it was 25 out of 69 (36% of total compounds) for wines aged in MLT barrels.

At 18 months of maturation, non-wood-related volatiles such as ethyl octanoate and ethyl hexanoate were the most prominent contributors to the fruity aroma profile among non-wood related volatiles. Ethyl octanoate showed OAVs of 54.15, 31.67, and 90.43 in wines matured in LT, MT, and MLT barrels, respectively, while ethyl hexanoate reached OAVs of 36.10, 26.69, and 52.01, respectively. Both compounds showed significantly higher OAVs in wines aged in MLT barrels compared to wines aged in LT and MT barrels. β-damascenone stood out amongst C13 norisoprenoids for its significantly high OAV in wines matured in MLT barrels, showing values of 65.35 in wines aged in MLT barrels versus 41.64 and 38.86 in LT and MT barrels, respectively.

At 18 months of ageing, wood-related volatiles such as eugenol and guaiacol stood out due to their highest OAV within the family of volatile phenols, regardless of the level of toasting. Eugenol showed OAVs of 10.90, 8.31, and 16.03 in wines matured in LT, MT, and MLT barrels, respectively, with wines from MLT barrels showing significantly high values. Similarly, guaiacol showed an OAV of 2.80 in wines aged in MLT barrels, which was significantly higher compared to LT and MT barrels. This is consistent with previous studies [7,9] showing increased guaiacol content in wines aged with oak alternatives with higher toasting levels, as observed in MLT barrels at 18 months.

Eugenol and guaiacol are formed almost exclusively by the degradation of lignin during the toasting process [1,29] and their production occurs during the secondary phase of heating after the formation of phenolic aldehydes [29]. Consequently, these compounds are more abundant when higher toasting levels are applied [29,35,37], as observed in wines aged in MLT barrels after 18 months of ageing. The vanillin, responsible for sweet and vanilla-like notes, also showed higher OAVs in wines aged in MLT barrels compared to those matured in LT and MT barrels.

Furfural, responsible for incense and almond aromas, was also present at higher concentrations in wines aged in MLT barrels at the 18-month ageing mark. This agrees with previous studies [7,9], which reported increased furfural levels in wines aged with oak alternatives with higher toasting intensities.

Lastly, lactones like *cis*-whiskey-lactone exhibited higher OAVs in wines from MLT barrels of 10.72 compared to 7.54 and 5.96 in the case of wines aged in LT and MT barrels, respectively. These results align with the findings of De Simón et al. [9], who reported higher concentrations of *cis*-whiskey-lactone in wines aged with heavily toasted chips, which may explain the elevated levels observed in MLT barrels after 18 months of ageing. Similarly, *trans*-whiskey-lactones were also found in higher concentrations in wines at the 18-month ageing period when MLT barrels were employed. This agrees with the findings of De Simón et al. [9], who reported increased *trans*-whiskey-lactone levels in wines aged with heavily toasted chips.

These results highlight the influence of toasting level on the evolution of volatile profiles, with LT barrels favouring short-term ageing and MLT barrels favouring long-term maturation. Having considered these findings, ageing wines in barrels with higher toasting levels reduces the concentration of non-oak-related volatiles during shorter ageing periods (12 months). This explains why wines aged in lightly toasted barrels (LT) exhibited the highest concentrations in most non-oak volatile families. However, after 18 months of ageing, wines in MLT barrels exhibited higher concentrations of non-oak-derived volatiles, followed by wines aged in MT and, finally, LT barrels.

It is also important to note that a decrease in the concentration of detectable compounds after 18 months, compared to 12 months, was observed. This reduction was likely due to the degradation of volatile compounds, particularly those not related to oak. The degradation could primarily be driven by oxidation processes within the barrel, as well as other complex chemical reactions, such as condensation and esterification, which continuously occur during the ageing process. These ongoing interactions lead to the transformation or reduction in certain volatile compounds, resulting in lower concentrations of specific compounds after prolonged ageing [1,3,7].

### 2.3. Grain Size Effect on Volatile Profile of Tempranillo Red Wine Aged in Quercus petraea Barrels

Table 3 shows the volatile concentration (µg 4-nonanol/L) and the odour active value (OAV) for each volatile compound in wines after 12 and 18 months of barrel ageing according to the grain size employed [standard grain (SG) and extra fine grain (EG)].

A notable difference influenced by both the grain type and the ageing period was observed. In fact, for most volatiles, there was a higher concentration in wines aged in SG barrels for 12 months and in wines aged in EG barrels for 18 months.

At 12 months of ageing, wines aged in SG barrels showed higher concentrations of all volatile families compared to those matured in EG barrels, and it was more markedly in furanic compounds and vanillin, where wines aged in SG barrels showed concentrations of 270% and 175%, respectively, higher than those found in wines aged in EG barrels. Specifically, wines aged in SG barrels for 12 months showed concentrations of furfural, 5-methylfurfural, and furfuryl alcohol that were 195%, 342%, and 357% higher, respectively, than those obtained in wines aged in EG barrels for 12 months. Similarly, C6 alcohols, higher alcohols, C13-norisoprenoids, ethyl esters and acetates, fatty acids, γ-lactones, and carbonyl compounds showed higher concentrations in wines aged in SG, with percentage increases ranging from 41% to 60%. Volatile phenols also displayed a significant increase of 64% in wines aged in SG barrels, and lactones showed a 27.04% increase.

At 12 months of ageing, 26 out of 69 volatile compounds (38% of total compounds) were found at average concentrations higher than their corresponding odour thresholds (OAV > 1) in wines aged in SG barrels. Wines matured in EG barrels showed 23 out of 69 quantified volatile compounds (33% of total compounds) with concentrations higher than their corresponding odour thresholds (OAV > 1). Among non-wood-related volatiles, esters, such as ethyl octanoate and ethyl hexanoate, were the most prominent contributors to the fruity aroma profile. Ethyl octanoate showed an OAV of 88.09 in wines aged in SG barrels and 64.05 in wines matured in EG barrels, while ethyl hexanoate reached an OAV of 43.46 in wines aged in SG barrels versus 30.53 in wines aged in EG barrels. The higher OAV in SG-aged wines compared to EG-matured wines could be due to the lower oxygen permeability of larger grain barrels, which would allow for a lower evaporation of water and, thus, a decrease in esterification reactions in accordance with the law of mass action [38]. β-damascenone stood out among C13 noirsoprenoids for its highest OAV, showing values of 60.61 in wines aged in SG barrels versus 42.55 in wines aged in EG barrels. The significantly higher OAV in SG-matured wines suggested that the lower oxidative environment provided by SG barrels enhanced the stability of this compound, whereas the higher oxygen permeation in EG barrels promoted oxidative processes that lead to a greater loss of volatile compounds [37]. Among the higher alcohols, 2-phenylethanol, characterised by its rose-like and floral aromas, showed an OAV of 8.15 in SG wines versus 5.27 in EG wines.

Among the wood-related volatiles, eugenol and guaiacol stood out by their high OAV. Eugenol and guaiacol showed a higher significant OAV in wines aged in SG barrels (15.5 and 3.07, respectively) compared to wines aged in EG barrels (8.85 and 1.15, respectively). These differences are not consistent with the results found in a study by Bosso et al. which reported no significant differences in the guaiacol or eugenol content of wines aged in fine and extra fine grain barrels, but it is worth noting that the ageing time was only 6 months [11].

Vanillin also showed a higher OAV in wines ageing in SG barrels (1.65) compared to wines maturing in EG barrels (0.60). This increase in wines from SG barrels was likely due to the greater oxidative degradation of vanillin in smaller-grain wood during barrel ageing. The enhanced concentration of these compounds in SG-matured wines could contribute significantly to their aromatic richness and complexity. These findings indicated the role of larger grain barrels in enhancing the aromatic intensity and complexity of wines after 12 months of maturation.

The analysis of volatile compound after 18 months varied compared to 12 months of ageing. While wines maturing in SG barrels continued showing higher concentrations of furanic compounds (78%) and vanillin (40%), certain differences narrowed or reversed, reflecting dynamic interactions between barrel grain type and extended maturation. The concentrations of C6 alcohols, higher alcohols, C13-norisoprenoids, ethyl esters and acetates, fatty acids, and lactones were higher in EG barrels than in SG barrels, ranging from 17% to 39% higher. Carbonyl compounds were also found in higher concentrations in EG barrels, with levels being 87% higher compared to SG barrels. γ-lactones and volatile phenols, which showed significant differences at 12 months, presented no statistically significant variation at 18 months. These findings demonstrated that the impact of barrel grain type on the evolution of volatile compounds became more pronounced with prolonged maturation, as certain compound families exhibited substantial increases, while others remain unchanged or even declined. Additionally, some initially significant differences lost statistical significance over time. This underscores the necessity of accounting for both barrel characteristics and ageing duration to achieve the desired wine profile.

At 18 months of maturation, 24 out of 69 volatile compounds (35% of total compounds) were found at average concentrations higher than their corresponding odour thresholds (OAV > 1) in wines aged in SG barrels. Wines matured in EG barrels showed 23 out of 69 quantified volatile compounds (33% of total compounds) with concentrations higher than their corresponding odour thresholds (OAV > 1). Unlike what was observed after 12 months of ageing, wines aged in EG barrels for 18 months showed significantly higher OAVs for most volatile compounds compared to those matured in SG barrels, as reflected in their total OAV of 343, compared to 262 for SG wines. This increase was particularly evident in β-ionone, ethyl butyrate, and *cis*-whiskey-lactone, in which EG aged wines exhibited a higher OAV than in SG-matured wines. It should be noted that the highest difference was observed in *cis*-whiskey-lactone, with an OAV more than doubling in wines from EG barrels. Overall, the higher total OAV in wines from EG barrels at 18 months of maturation underscored their potential to enhance the aromatic profile of aged wines.

These results showed the dynamic evolution of volatile compounds during wine ageing, with SG barrels providing greater aromatic complexity at 12 months of ageing, while EG barrels progressively enhanced certain fruity and coconut sweet notes after 18 months.

### 2.4. Combined Effect of Toasting Level and Grain Sizes on Volatile Profile of Tempranillo Red Wine Aged in Quercus petraea Barrels

Table 4 and Table 5 show the combined effect of toasting [light toasting (LT), medium toasting (MT), and medium long toasting (MLT)] and grain size [standard grain (SG), and extra fine grain (EG)] at 12 and 18 months of ageing. Wines aged in LT and SG barrels had the highest concentrations for most volatiles at the 12-month ageing period while wines matured in MLT and EG barrels had the highest concentrations after 18 months of the ageing period.

After 12 months of barrel ageing (Table 4), all volatile families were present in higher concentrations in wines aged in LT × SG barrels, except for the furanic compound family. For this family, LT × SG and MT × SG showed comparable levels, followed by MLT × SG. Overall, regardless of the toasting degree, wines aged in standard grain (SG) barrels exhibited higher concentrations than those found in wines matured in extra fine grain (EG) barrels. The lower concentrations observed in EG barrels could be attributed to their smaller pores, which promoted greater oxidation.

After 18 months of ageing (Table 5), MLT × EG barrels showed the highest concentrations of most volatile compounds. However, the effect of grain size at 18 months of ageing was not as clear as at 12 months. In this regard, under light toasting, wines aged in barrels with SG yielded higher concentrations of β-pinene, citral, furfural, 5-methylfurfural, furfuryl alcohol, and vanillin compared to EG barrels. In the same way, wines matured in MT × SG barrels showed higher concentrations of oak-related compounds, such as furfural, 5-methylfurfural, furfuryl alcohol, *trans*-whiskey-lactone, guaiacol, and vanillin, compared to wines aged in MT × EG barrels. However, wines matured in MLT barrels exhibited higher volatile concentrations in EG compared to SG. This phenomenon, which was observed at the 18 months of ageing, could have been due to the oxygen transference provided by the barrel decrease over time [37], since the wood micropores in the extra fine grain barrel could become progressively obstructed during extended ageing periods. In contrast, the larger pores in standard grain barrels allowed for continued oxygen ingress, resulting in the degradation of volatile compounds. The fact that this only occurs in MLT barrels at the 18-month ageing period might be due to the clogging being related to the degree of toasting, with lower toasting levels seemingly unaffected, and the maturation duration, as this phenomenon was observed only at 18 months.

These results indicated that after 12 months of ageing, higher volatile complexity was found in wines aged in light toasting (LT) and standard sized grain (SG) barrels, whereas for a longer maturation period (18 months), medium long toasting (MLT) and extra fine grain sized (EG) barrels produced wines with higher volatile concentrations. Specifically, at 12 months of maturation, SG barrels produced wines with higher volatile concentrations when than EG barrels, particularly when light toasted barrels were used. MT × SG wines were distinguished by a pronounced presence of floral, incense, spice, almond, and vanilla flavours. In contrast, MLT × SG wines showed nuttier, woody, coconut, vanilla, clove, and smoky aromas. At 18 months of ageing, wines aged in SG barrels exhibited more pronounced aromas of pine, hay, lemon, incense, almonds, spice, and vanilla when light toasting (LT) was used. When medium toasting (MT) was employed, these wines developed additional notes of wood, coconut, smoky, and toast aromas. However, for medium long toasting (MLT), it was the EG barrels that produced wines with higher concentrations of most of the 69 volatiles analysed. Therefore, these results underscore the importance of tailoring barrel characteristics to achieve specific aromatic profiles. These insights confirmed that the synergistic effects of toasting and grain size provide winemakers with a nuanced approach to controlling wine maturation outcomes.

**Figure 1:**
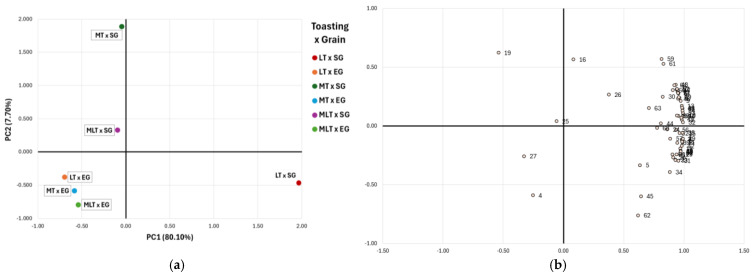
(**a**) Classification of wines after 12 months of barrel ageing by principal component analysis (PCA) according to volatile compounds analysed. (**b**) Contribution of variables in coordinate system defined by first two principal components (loading plot). LT: light toasting; MT: medium toasting; MLT: medium long toasting; SG: standard grain size; EG: extra fine grain size. 1: 1-hexanol; 2: Z-3-hexenol; 3: E-3-hexenol; 4: E-2-hexenol; 5: Z-2-hexenol; 6: 1-propanol; 7: 2-octanol; 8: isobutanol; 9: 1-butanol; 10: isoamyl alcohol; 11: 3-methyl-1-pentanol; 12: 2,3-butanediol; 13: 3-methyltiopropanol; 14: benzyl alcohol; 15: 2-phenylethanol; 16: α-ionone; 17: β-ionone; 18: β-damascenone; 19: Terpinolene; 20: α-terpineol; 21: *trans*-geraniol; 22: *cis*-geraniol; 23: β -citronellol; 24: linalool; 25: β-pinene; 26: 4-carvomenthenol; 27: citral; 28: ethyl butyrate; 29: Ethyl 2-methylbutyrate; 30: ethyl decanoate; 31: ethyl isovalerate; 32: ethyl myristate; 33: methyl salicylate; 34: hexyl acetate; 35: methyl vanillate; 36: ethyl vanillate; 37: ethyl hexanoate; 38: ethyl lactate; 39: ethyl octanoate; 40: diethyl succinate; 41: isoamyl acetate; 42: 2-phenylethyl acetate; 43: propanoic acid; 44: geranic acid; 45: nerolic acid; 46: pentanoic acid; 47: isobutyric acid; 48: 2-methylbutyric acid; 49: hexanoic acid; 50: octanoic acid; 51: butyric acid; 52: acetic acid; 53: isovaleric acid; 54: γ-butyrolactone; 55: acetoin; 56: benzaldehyde; 57: hexanal; 58: vanillyl acetone; 59: furfural; 60: 5-methylfurfural; 61: furfuryl alcohol; 62: *cis*-whiskey-lactone; 63: *trans*-whiskey-lactone; 64: 4-vinylphenol; 65: 4-vinylguaiacol; 66: eugenol; 67: guaiacol; 68: 4-ethylphenol; 69: vanillin.

**Figure 2:**
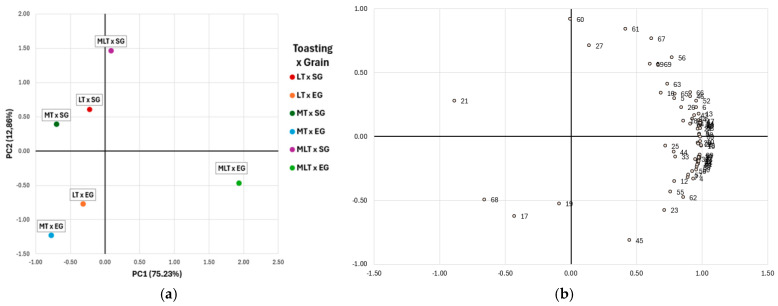
(**a**) Classification of wines after 18 months of barrel ageing by principal component analysis (PCA) according to volatile compounds analysed. (**b**) Contribution of variables in coordinate system defined by first two principal components (loading plot). LT: light toasting; MT: medium toasting; MLT: medium long toasting; SG: standard grain size; EG: extra fine grain size. 1: 1-hexanol; 2: Z-3-hexenol; 3: E-3-hexenol; 4: E-2-hexenol; 5: Z-2-hexenol; 6: 1-propanol; 7: 2-octanol; 8: isobutanol; 9: 1-butanol; 10: isoamyl alcohol; 11: 3-methyl-1-pentanol; 12: 2,3-butanediol; 13: 3-methyltiopropanol; 14: benzyl alcohol; 15: 2-phenylethanol; 16: α-ionone; 17: β-ionone; 18: β-damascenone; 19: Terpinolene; 20: α-terpineol; 21: *trans*-geraniol; 22: *cis*-geraniol; 23: β -citronellol; 24: linalool; 25: β-pinene; 26: 4-carvomenthenol; 27: citral; 28: ethyl butyrate; 29: Ethyl 2-methylbutyrate; 30: ethyl decanoate; 31: ethyl isovalerate; 32: ethyl myristate; 33: methyl salicylate; 34: hexyl acetate; 35: methyl vanillate; 36: ethyl vanillate; 37: ethyl hexanoate; 38: ethyl lactate; 39: ethyl octanoate; 40: diethyl succinate; 41: isoamyl acetate; 42: 2-phenylethyl acetate; 43: propanoic acid; 44: geranic acid; 45: nerolic acid; 46: pentanoic acid; 47: isobutyric acid; 48: 2-methylbutyric acid; 49: hexanoic acid; 50: octanoic acid; 51: butyric acid; 52: acetic acid; 53: isovaleric acid; 54: γ-butyrolactone; 55: acetoin; 56: benzaldehyde; 57: hexanal; 58: vanillyl acetone; 59: furfural; 60: 5-methylfurfural; 61: furfuryl alcohol; 62: *cis*-whiskey-lactone; 63: *trans*-whiskey-lactone; 64: 4-vinylphenol; 65: 4-vinylguaiacol; 66: eugenol; 67: guaiacol; 68: 4-ethylphenol; 69: vanillin.

Principal component analysis (PCA) was performed on the concentration of the wine volatile compounds analysed after 12 and 18 months of barrel ageing (Figure 1 and Figure 2, respectively), which allowed differentiating the wines according to the levels of toasting and types of grains used in combination.

Figure 1 shows the distribution of the wines after 12 months of barrel maturation and the volatile compound loads. The first two principal components explained 87.80% of the cumulative variance. Principal Component 1 (PC1), which explained 80.10% of the variance, was strongly positively correlated with ethyl butyrate (factor loading = 0.972), β-damascenone (0.970), β-ionone (0.940), methyl salicylate (0.928), α-terpineol (0.911), eugenol (0.906), and *cis*-whiskey-lactone (0.619) among others. PC1 separated wines from LT × SG barrels from the rest, highlighting their distinct characteristics. As previously detailed (Table 4), wines aged in LT × SG barrels exhibited higher levels of these volatile compounds compared to wines aged in other barrels, which displayed lower concentrations.

On the contrary, Principal Component 2 (PC2) accounted for only 7.70% of the variance. It was positively correlated with terpinolene (0.624) and furfural (0.572) and negatively correlated with E-2-hexenol (−0.590), *cis*-whiskey-lactone (−0.761), and nerolic acid (−0.598). PC2 primarily separated the wines based on grain size (SG and EG), with the exception of LT × SG wines, which were positioned on the negative side of PC2 alongside wines aged in EG barrels. Wines aged in MT × SG barrels were located in the upper part of the graph, with the most positive value in PC2 and close to zero in PC1. This indicates that these wines were characterised by variations that were more associated with PC2 than with PC1. In fact, wines aged in MT × SG barrels showed higher contents of terpinolene and furfural and lower contents of E-2-hexenol, *cis*-whiskey-lactone, and nerolic acid than wines located in the most negative part of the PCA space (MLT × EG), which is in agreement with the results shown in Table 4.

Figure 2 shows the distribution of the wines after 18 months of barrel ageing and the volatile compound loads. The two first principal components explained 88.09% of the data variation. PC1 explained 75.23% of the variability and was positively correlated with Z-3-hexenol (factor loading = 0.983), isoamyl alcohol (0.989), 2-phenylethanol (0.995), ethyl decanoate (0.988), diethyl succinate (0.995), γ-butyrolactone (0.988), *cis*-whiskey-lactone (0.857), and eugenol (0.910). PC1 allowed us to distinguish between wines from barrels with MLT and the other two toasting levels (LT and MT). Wines aged in MLT × EG barrels were widely separated from wines from MLT × SG barrels, although both were placed on the positive side of PC1. Therefore, and as it was previously explained (Table 5), wines aged in MLT × SG barrels showed a higher content of the abovementioned volatile compounds, contrarily to the wines aged in the other barrels, which showed, in general, a lower content of these compounds. On the other hand, PC2 only explained 12.86% of the data variation, but it was positively correlated to 5-methylfurfural (0.917) and furfuryl alcohol (0.843), and negatively to nerolic acid (−0.815). PC2 mainly separated the wines according to grain size (SG and EG). Wines aged in SG barrels showed positive values for this PC, and thus a higher content of 5-methylfurfural and furfuryl alcohol, and a lower content of nerolic acid, which is in agreement with results shown in Table 5.

## 3. Materials and Methods

### 3.1. Barrels

New 225 L *Quercus petraea* barrels were used in this study. The barrels were made by the Ermitage Cooperage (Bourgogne, France) in 2018. A total of 36 barrels were used in the investigation with three different types of toasting (light toasting—LT; medium toasting—MT; and medium long toasting—MLT), 12 barrels in total for each toasting type and with two different grain types (standard grain—SG; extra fine grain—EG), and 18 barrels in total for each grain type. Therefore, a total of six barrels were analysed for each combination (LT × SG, LT × EG, MT × SG, MT × EG, MLT × SG, and MLT × EG). Table 6 shows the experimental design that was carried out.

The toasting was conducted by traditional toasting with temperature control by oak fire.

### 3.2. Wine Barrel Ageing and Sample Collection

Red Tempranillo wine was produced in the harvest of 2018 using the traditional red vinification method at Bodegas Ramón Bilbao S.A. located in Haro, La Rioja (Spain).

The wines were placed in the barrels in March 2019, where they underwent malolactic fermentation (MLF) and where they aged in 36 new 225 L *Quercus petraea* barrels. Sampling of the wine was conducted at 12 months (March 2020) and 18 months (September 2020) of barrel ageing. The volatile composition was analysed on the 36 barrels in two different moments to then see the toasting effect (12 barrels for each toasting level) and the grain type effect (18 barrels for each grain type) on the analysed compounds. The experimental design involved 36 barrels distributed according to the toasting level and grain size (Table 6). One sample was taken from each barrel.

### 3.3. Wine Volatile Composition

The volatile compounds in the wine were analysed using gas chromatography–mass spectrometry (GC-MS) after liquid–liquid extraction [39]. The analyses were made in triplicate.

GC analysis was performed using an Agilent GC 7890 N chromatograph (Agilent Technologies, Palo Alto, CA, USA) coupled to an Agilent 7000 C, a capillary column coated with CP-Wax 52 CB (50 m × 0.25 mm i.d., 0.2 μm film thickness, Chrompack, São Paulo, Brazil), and the conditions described in [39].

The volatile compounds were identified using Mass Hunter WorkStation Software (Agilent Mass Hunter Qualitative Analysis B.07.00) and NIST 2.2. The concentration of the pure standard compounds was matched to that of the wine samples (μg/L). Semi-quantitative data were acquired by calculating the relative peak area in relation to the internal standard (4-nonanol).

### 3.4. Odour Active Values (OAVs)

The OAVs were calculated to establish, quantitatively, the contribution of each volatile compound to the aroma of the studied wines. This value was calculated using the following equation consulted in the scientific literature: OAV = concentration (μg/L)/odour threshold (µg/L) [40,41]. If the compounds were present at above-threshold levels (OAV > 1), these compounds were recognised to have the potential to actively contribute to the aroma of the wines [42,43]. If the OAVs were lower than 1 but higher than 0.1, these compounds could also potentially contribute to the wine aromas by additive effects [42]. But if their OAVs were lower than 0.1, their effects on wine aromas could be negligible [44,45].

Table 7 displays the odour thresholds and aromatic descriptors for the analysed volatiles.

### 3.5. Statistical Analysis

The statistical analyses were performed using SPSS Statistics 26 (IBM Corp., Armonk, NY, USA). A multivariate analysis of variance (MANOVA) was conducted to examine the effects of the toasting level and grain size and their interaction on all the measured volatile compounds in the wines. Pillai’s trace was used as the test statistics for the MANOVA analysis. Principal component analysis was employed to explore potential differentiation among the wines according to the type of toasting and grain size applied. Statistical significance among the treatments was determined using the Duncan’s test, with *p* ≤ 0.05 considered as the threshold for significance. The experimental unit consisted of a total of 36 oak barrels, distributed across three toasting levels (12 barrels per toasting level) and two grain sizes (18 barrels per grain size). The data presented in all the tables originates from the dataset of the 36 barrels.

## 4. Conclusions

This study demonstrates that both toasting level and grain size significantly influenced the volatile composition of Tempranillo red wines aged in *Quercus petraea* barrels over 12 and 18 months, with the toasting level exerting a more pronounced effect. For shorter ageing periods, lighter toasting combined with standard grain barrels (LT × SG) yielded higher concentrations for all volatile families in wines, except for the furanic compound family. For longer maturation periods, medium long toasting combined with extra fine grain barrels (MLT × EG) yielded higher concentrations for most volatile compounds in wines.

While grain size had a lower impact, toasting level consistently demonstrated a stronger influence on the aromatic profile of the aged wines, suggesting that thermal degradation of wood macromolecules during toasting plays a pivotal role in determining wine volatile composition.

The different influence of the grain and toast of the barrel and their combination on the volatile compounds at 12 and 18 months offers winemakers valuable tools to tailor the aromatic complexity of wines. By aligning barrel specifications with the desired ageing periods and sensory outcomes, greater control over wine maturation can be achieved.

The evolution of volatile compounds between 12 and 18 months highlights the dynamic interplay between wood characteristics and wine maturation. This underscores the need for winemakers to consider temporal aspects when designing ageing regimens.

## Figures and Tables

**Table 1:** Multivariate analysis of variance (MANOVA) of volatile composition of Tempranillo red wines aged in *Quercus petraea* barrels. Percentage of attributable variance (%) of independent effect of toasting level and grain size of barrel, and interaction of both (toasting level × grain size).

	12 Months							18 Months						
	Toasting Level (%)	Sig.	Grain Size (%)	Sig.	Interaction (%)	Sig.	Error (%)	Toasting Level (%)	Sig.	Grain Size (%)	Sig.	Interaction (%)	Sig.	Error (%)
**C6 Alcohols**	57.88	**	5.63	ns	3.43	ns	33.05	36.85	**	16.32	**	26.40	**	20.35
1-hexanol	55.65	**	5.92	ns	3.57	ns	34.87	36.50	**	16.38	**	26.63	**	20.50
Z-3-hexenol	64.94	**	2.47	ns	2.30	ns	30.29	39.72	**	15.12	*	24.00	*	21.16
E-3-hexenol	68.71	***	2.81	ns	1.78	ns	26.70	38.83	**	15.64	*	25.11	**	20.42
E-2-hexenol	24.69	***	17.34	***	54.70	***	3.27	35.52	***	33.08	***	27.94	***	3.45
Z-2-hexenol	98.84	***	0.83	***	0.22	**	0.11	80.36	***	0.03	ns	10.27	*	9.33
**Higher alcohols**	65.45	***	4.74	ns	2.88	ns	26.92	50.86	***	10.76	*	22.33	**	16.05
1-propanol	40.89	***	25.05	***	25.01	***	9.05	72.15	***	1.16	ns	11.83	*	14.86
2-octanol	45.68	***	7.69	ns	26.99	**	19.64	21.70	ns	17.31	*	18.24	ns	42.75
Isobutanol	70.92	***	8.39	*	5.06	ns	15.62	45.93	***	4.79	ns	28.48	**	20.80
1-butanol	70.12	***	8.92	*	5.73	ns	15.23	61.38	***	2.79	ns	22.18	**	13.66
Isoamyl alcohol	70.51	***	1.71	ns	3.21	ns	24.56	45.62	***	7.72	*	28.93	**	17.73
3-methyl-1-pentanol	56.45	**	4.96	ns	4.68	ns	33.91	38.19	**	15.41	*	23.46	*	22.94
2.3-butanediol	29.54	**	20.52	**	36.22	ns	13.72	45.42	***	47.66	***	2.03	ns	4.89
3-methylthiopropanol	80.51	***	0.35	ns	3.14	ns	16.01	67.69	***	1.87	ns	18.20	**	12.25
Benzyl alcohol	74.29	***	2.00	ns	2.65	ns	21.06	59.25	***	3.77	ns	21.59	**	15.39
2-phenylethanol	54.82	**	5.98	ns	5.08	ns	34.11	51.43	***	12.45	*	18.52	*	17.60
**C_13_-norisoprenoids**	41.19	***	42.75	***	3.43	ns	12.63	68.65	***	9.29	*	1.89	ns	20.17
α-ionone	32.67	***	45.40	***	18.58	***	3.35	77.37	***	0.51	ns	10.36	*	11.75
β-ionone	23.51	***	58.54	***	15.21	***	2.74	18.00	***	26.75	***	51.31	***	3.94
β-damascenone	62.63	***	8.00	ns	3.14	ns	26.23	66.42	***	5.83	ns	0.10	ns	27.65
**Terpenes**	70.95	***	14.53	**	4.06	ns	10.46	32.85	ns	0.36	ns	15.00	ns	51.79
Terpinolene	16.90	***	55.47	***	27.43	***	0.20	41.27	***	39.81	***	18.20	***	0.72
α-terpineol	42.51	***	21.75	**	21.48	**	14.26	51.41	***	9.53	*	26.43	**	12.63
E-geraniol	44.42	***	28.42	***	13.25	*	13.91	40.01	***	21.56	***	29.48	***	8.95
Z-geraniol (Nerol)	68.90	***	0.08	ns	6.33	ns	24.70	46.01	***	8.06	*	30.20	**	15.73
β-citronellol	54.54	***	3.55	*	36.77	***	5.14	19.80	***	55.40	***	20.78	***	4.02
Linalool	77.61	***	4.04	*	10.51	**	7.83	57.58	***	4.49	ns	19.55	*	18.37
β-pinene	43.41	***	6.86	**	41.27	***	8.46	23.37	***	1.81	*	71.92	***	2.90
4-carvomenthenol	18.27	**	4.23	*	69.03	***	8.47	81.53	***	2.54	ns	1.71	ns	14.22
Citral	26.45	***	45.52	***	21.02	***	7.01	28.64	***	37.85	***	28.18	***	5.32
**Ethyl esters and acetates**	71.29	***	8.77	**	9.53	*	10.41	50.10	***	7.71	*	25.62	**	16.56
Ethyl butyrate	56.96	**	3.59	ns	3.63	ns	35.81	35.26	**	17.34	**	27.45	**	19.95
Ethyl 2-methylbutyrate	64.26	***	3.07	ns	3.23	ns	29.43	40.27	**	13.96	*	24.66	*	21.11
Ethyl decanoate	43.56	***	16.43	**	23.81	**	16.20	65.73	***	4.73	*	18.33	**	11.20
Ethyl isovalerate	62.92	***	0.03	ns	8.56	ns	28.50	34.16	**	15.91	*	25.32	*	24.61
Ethyl myristate	35.87	***	19.61	**	31.61	***	12.91	38.04	**	13.21	*	28.05	**	20.71
Methyl salicylate	38.03	***	23.21	***	35.95	***	2.82	60.25	***	12.05	**	17.36	**	10.35
Hexyl acetate	74.56	***	7.97	**	7.76	*	9.72	56.72	***	2.44	ns	31.09	***	9.74
Methyl vanillate	68.04	***	5.87	ns	0.65	ns	25.44	31.02	**	17.65	*	26.24	*	25.09
Ethyl vanillate	64.63	***	2.65	ns	4.91	ns	27.81	37.72	**	14.13	*	29.10	**	19.06
Ethyl hexanoate	59.90	**	4.21	ns	2.73	ns	33.16	38.38	**	14.58	*	28.30	**	18.75
Ethyl lactate	66.56	***	9.22	*	9.29	ns	14.93	45.34	**	5.04	ns	29.13	**	20.49
Ethyl octanoate	51.69	**	6.91	ns	4.00	ns	37.40	49.11	***	7.01	*	27.02	**	16.86
Diethyl succinate	58.37	**	3.87	ns	7.64	ns	30.13	51.89	***	12.02	*	16.56	*	19.53
Isoamyl acetate	52.64	**	6.02	ns	2.10	ns	39.25	35.48	**	17.93	**	24.07	*	22.52
2-phenylethyl acetate	52.44	**	3.69	ns	3.31	ns	40.56	37.24	**	16.61	**	25.33	**	20.82
**Fatty acids**	61.06	***	13.07	**	10.63	*	15.24	46.71	***	11.68	**	27.74	**	13.87
Propanoic acid	38.04	***	18.90	**	29.29	**	13.77	63.22	***	2.16	ns	16.91	*	17.70
Geranic acid	33.04	***	3.77	***	60.79	***	2.40	63.02	***	8.13	**	23.41	***	5.44
Nerolic acid	67.79	***	1.64	ns	1.87	ns	28.70	0.44	ns	59.20	***	11.34	ns	29.01
Pentanoic acid	83.46	***	0.12	ns	6.48	*	9.93	78.17	***	0.43	ns	3.15	ns	18.26
Isobutyric acid	57.74	**	0.06	ns	13.06	ns	29.14	57.70	***	2.74	ns	22.53	**	17.03
2-methylbutyric acid	55.07	***	13.55	**	14.46	*	16.92	57.59	***	3.71	ns	24.32	**	14.38
Hexanoic acid	52.00	**	5.55	ns	4.80	ns	37.65	33.94	**	17.92	**	28.67	**	19.47
Octanoic acid	55.46	**	2.94	ns	5.59	ns	36.02	32.24	**	21.01	**	29.67	**	17.08
Butyric acid	73.58	***	6.01	*	7.63	ns	12.78	50.14	***	2.97	ns	28.96	**	17.93
Acetic acid	34.71	***	29.67	***	24.15	**	11.47	72.26	***	0.24	ns	15.92	**	11.58
Isovaleric acid	62.45	***	11.49	*	9.81	ns	16.25	54.06	***	4.20	ns	25.57	**	16.17
**γ-Lactones**	63.61	***	9.33	*	10.29	ns	16.77	62.61	***	3.72	ns	20.04	**	13.63
γ-butyrolactone	63.61	***	9.33	*	10.29	ns	16.77	62.61	***	3.72	ns	20.04	**	13.63
**Carbonyl compounds**	81.62	***	4.08	ns	0.53	ns	13.77	23.13	***	32.26	***	35.62	***	8.99
Acetoin	81.62	***	4.08	ns	0.53	ns	13.77	23.13	***	32.26	***	35.62	***	8.99
**Others**	64.72	***	10.66	**	12.37	*	12.25	35.98	***	16.92	***	40.01	***	7.10
Benzaldehyde	69.78	***	8.73	**	11.65	**	9.85	66.43	***	7.22	ns	5.33	ns	21.02
Hexanal	44.93	***	20.30	**	20.66	**	14.11	24.23	**	21.76	**	39.53	***	14.48
Vanillyl acetone	43.67	**	12.70	*	16.79	ns	26.84	28.57	***	21.10	***	40.35	***	9.98
**Furanic compounds**	4.68	ns	76.78	***	5.46	***	13.08	25.01	*	38.46	***	14.39	*	22.14
Furfural	4.68	ns	72.65	***	7.55	***	15.12	26.97	*	10.96	*	36.84	**	25.23
5-methylfurfural	5.94	ns	62.80	***	7.26	***	24.00	3.96	ns	72.84	***	6.40	ns	16.80
Furfuryl alcohol	5.02	ns	82.23	***	3.28	***	9.46	47.89	**	29.17	**	0.77	ns	22.17
**Lactones**	51.54	**	2.65	ns	14.14	ns	31.67	56.98	***	17.66	**	8.89	ns	16.47
*cis*-whiskey-lactone	47.47	**	18.52	**	11.84	ns	22.17	26.28	**	45.56	***	14.43	*	13.73
*trans*-whiskey-lactone	53.94	***	13.29	*	10.51	ns	22.26	69.94	***	0.24	ns	18.63	**	11.20
**Volatile phenols**	63.16	***	11.06	*	3.99	ns	21.80	76.88	***	1.25	ns	6.93	ns	14.94
4-vinylphenol	39.53	**	27.79	**	6.21	ns	26.47	68.56	***	7.51	*	6.38	ns	17.55
4-vinylguaiacol	71.32	***	4.85	ns	0.36	ns	23.47	77.89	***	0.46	ns	7.72	ns	13.93
Eugenol	63.67	***	6.99	ns	7.53	ns	21.81	63.49	**	0.07	ns	6.51	ns	29.93
Guaiacol	56.11	**	3.58	ns	5.55	ns	34.76	60.57	***	16.50	**	2.27	ns	20.66
4-ethylphenol	85.54	***	0.72	ns	0.69	ns	13.05	85.66	***	2.97	ns	1.94	ns	9.43
**Phenolic aldehydes**	65.38	***	9.21	*	7.09	ns	18.31	41.14	***	13.78	**	34.22	***	10.87
Vanillin	65.38	***	9.21	*	7.09	ns	18.31	41.14	***	13.78	**	34.22	***	10.87

The stars indicate the level of significant differences at 95% (*), 99% (**), and 99.9% (***). ns indicates that no significant differences were found.

**Table 2:** Concentration (µg 4-nonanol/L) and odour active values (OAVs) of volatile compounds in wines after 12 and 18 months of ageing. MANOVA statistical analysis and effect of toasting level on Tempranillo red wines aged in *Quercus petraea* oak barrels.

	12 Months										18 Months									
	LT			MT			MLT				LT			MT			MLT			
	µg/L		OAV	µg/L		OAV	µg/L		OAV	Sig.	µg/L		OAV	µg/L		OAV	µg/L		OAV	Sig.
**C6 Alcohols**	1698	b	0.53	1117	a	0.32	1199	a	0.36	**	1414	a	0.40	1085	a	0.31	1992	b	0.58	**
1-hexanol	1566	b	0.20	1043	a	0.13	1158	a	0.14	*	1320	a	0.16	1013	a	0.13	1854	b	0.23	**
Z-3-hexenol	79.36	b	0.20	54.07	a	0.14	59.45	a	0.15	**	67.61	a	0.17	51.91	a	0.13	96.57	b	0.24	**
E-3-hexenol	27.27	b	0.07	18.10	a	0.05	20.79	a	0.05	**	22.92	a	0.06	17.18	a	0.04	33.73	b	0.08	**
E-2-hexenol	0.31	a	0.00	0.63	b	0.00	1.02	c	0.00	***	1.40	b	0.00	0.54	a	0.00	3.01	c	0.01	***
Z-2-hexenol	26.14	b	0.07	3.60	a	0.01	3.25	a	0.01	***	2.25	a	0.01	3.02	b	0.01	5.44	c	0.01	***
**Higher alcohols**	124,402	b	13.83	89,210	a	10.74	94,305	a	11.33	*	103,189	a	12.17	79,921	a	10.16	154,563	b	16.25	***
1-propanol	395.02	a	0.00	342.05	a	0.00	308.22	a	0.00	ns	350.51	a	0.00	262.79	a	0.00	607.19	b	0.00	***
2-octanol	488.11	b	4.07	464.82	a	3.87	473.12	ab	3.94	*	489.07	ab	4.08	468.43	a	3.90	498.19	b	4.15	ns
Isobutanol	2491	b	0.06	2158	ab	0.05	1899	a	0.05	ns	2190	a	0.05	1609	a	0.04	3264	b	0.08	***
1-butanol	119.07	b	0.00	99.91	ab	0.00	89.66	a	0.00	ns	104.01	a	0.00	79.56	a	0.00	166.85	b	0.00	***
Isoamyl alcohol	38,361	b	1.28	28,973	a	0.97	28,978	a	0.97	*	31,367	a	1.05	24,377	a	0.81	48,100	b	1.60	***
3-methyl-1-pentanol	59.64	b	0.00	39.23	a	0.00	43.68	a	0.00	**	49.33	a	0.00	37.24	a	0.00	67.39	b	0.00	**
2.3-butanediol	9.91	c	0.00	7.58	b	0.00	6.31	a	0.00	***	5.76	b	0.00	2.84	a	0.00	9.55	c	0.00	***
3-methylthiopropanol	202.35	b	0.20	153.88	a	0.15	146.95	a	0.15	*	156.45	b	0.16	113.57	a	0.11	257.16	c	0.26	***
Benzyl alcohol	117.09	b	0.00	84.06	a	0.00	83.83	a	0.00	*	85.35	a	0.00	69.58	a	0.00	130.75	b	0.00	***
2-phenylethanol	82,159	b	8.22	56,887	a	5.69	62,275	a	6.23	*	68,390	a	6.84	52,900	a	5.29	101,461	b	10.15	***
**C_13_-norisoprenoids**	5.44	b	90.41	3.01	a	51.95	3.26	a	56.58	***	3.19	a	54.00	3.08	a	51.49	4.81	b	82.50	***
α-ionone	0.38	a	4.22	0.48	ab	5.35	0.58	b	6.40	**	0.75	a	8.34	0.69	a	7.62	1.28	b	14.21	***
β-ionone	1.69	b	18.77	0.45	a	5.01	0.40	a	4.44	***	0.36	b	4.02	0.45	c	5.01	0.26	a	2.94	***
β-damascenone	3.37	b	67.42	2.08	a	41.59	2.29	a	45.73	**	2.08	a	41.64	1.94	a	38.86	3.27	b	65.35	***
**Terpenes**	101.10	b	4.35	79.69	a	3.40	79.32	a	3.44	*	80.63	ab	3.43	70.65	a	3.06	85.72	b	3.42	ns
Terpinolene	0.40	b	0.01	0.51	b	0.01	0.31	a	0.01	*	0.84	c	0.02	0.40	a	0.01	0.46	b	0.01	***
α-terpineol	6.62	b	0.01	4.34	a	0.00	4.57	a	0.00	*	4.69	b	0.00	3.59	a	0.00	5.90	c	0.01	***
E-geraniol	48.55	b	2.43	35.15	a	1.76	39.72	ab	1.99	*	38.72	b	1.94	33.15	b	1.66	19.86	a	0.99	***
Z-geraniol (Nerol)	35.26	b	1.76	30.05	ab	1.50	26.93	a	1.35	*	27.18	a	1.36	25.62	a	1.28	43.85	b	2.19	***
β-citronellol	2.25	c	0.02	0.77	a	0.01	1.46	b	0.01	***	1.28	a	0.01	1.77	b	0.02	2.55	c	0.03	***
Linalool	2.97	b	0.12	2.80	b	0.11	1.95	a	0.08	*	2.48	a	0.10	2.31	a	0.09	4.73	b	0.19	***
β-pinene	0.20	a	0.00	0.29	b	0.00	0.59	c	0.00	***	0.81	b	0.00	0.41	a	0.00	0.78	b	0.00	***
4-carvomenthenol	4.31	b	-	5.25	c	-	1.93	a	-	***	3.55	a	-	3.06	a	-	6.53	b	-	***
Citral	0.55	a	0.02	0.54	a	0.02	1.86	b	0.07	***	1.09	b	0.04	0.34	a	0.01	1.07	b	0.04	***
**Ethyl esters and acetates**	74,018	b	244.83	51,968	a	164.92	53,376	a	158.64	**	58,246	a	143.73	45,882	a	117.45	87,231	b	248.32	***
Ethyl butyrate	305.61	b	15.28	198.54	a	9.93	232.57	a	11.63	*	253.47	a	12.67	203.94	a	10.20	352.51	b	17.63	**
Ethyl 2-methylbutyrate	33.84	b	1.88	20.37	a	1.13	23.29	a	1.29	**	26.29	a	1.46	19.69	a	1.09	36.97	b	2.05	**
Ethyl decanoate	32.47	a	0.16	35.41	a	0.18	29.08	a	0.15	ns	13.30	a	0.07	10.81	a	0.05	29.04	b	0.15	***
Ethyl isovalerate	63.45	b	21.15	36.18	a	12.06	42.95	a	14.32	**	48.50	a	16.17	36.79	a	12.26	66.53	b	22.18	**
Ethyl myristate	15.39	b	0.01	11.73	a	0.01	11.07	a	0.01	**	9.20	a	0.00	9.93	a	0.00	17.19	b	0.01	**
Methyl salicylate	4.42	b	44.18	2.59	a	25.90	2.24	a	22.42	***	0.77	a	7.72	2.29	b	22.85	4.11	c	41.14	***
Hexyl acetate	277.18	b	0.41	130.14	a	0.19	152.12	a	0.23	***	188.54	b	0.28	93.60	a	0.14	296.08	c	0.44	***
Methyl vanillate	8.76	b	0.00	6.50	a	0.00	6.73	a	0.00	ns	7.17	a	0.00	5.53	a	0.00	9.86	b	0.00	**
Ethyl vanillate	530.84	b	0.54	331.76	a	0.34	391.69	a	0.40	*	400.93	a	0.40	324.03	a	0.33	532.11	b	0.54	**
Ethyl hexanoate	677.39	b	48.38	417.45	a	29.82	459.03	a	32.79	**	505.39	a	36.10	373.67	a	26.69	728.07	b	52.01	**
Ethyl lactate	43,498	b	0.28	33,759	a	0.22	32,014	a	0.21	*	35,675	a	0.23	27,168	a	0.18	52,989	b	0.34	***
Ethyl octanoate	468.97	b	93.79	365.64	a	73.13	306.42	a	61.28	*	270.77	b	54.15	158.35	a	31.67	452.13	c	90.43	***
Diethyl succinate	27,578	b	4.60	16,310	a	2.72	19,311	a	3.22	**	20,441	a	3.41	17,136	a	2.86	31,120	b	5.19	***
Isoamyl acetate	411.34	b	13.71	270.32	a	9.01	311.29	ab	10.38	*	321.61	a	10.72	264.93	a	8.83	471.31	b	15.71	**
2-phenylethyl acetate	111.93	b	0.45	71.67	a	0.29	81.73	a	0.33	*	83.72	a	0.33	73.24	a	0.29	126.57	b	0.51	**
**Fatty acids**	4856	b	9.78	3528	a	7.32	3711	a	7.24	*	3912	a	7.54	3115	a	6.37	5672	b	11.82	***
Propanoic acid	21.49	b	0.00	18.83	ab	0.00	16.30	a	0.00	ns	21.23	b	0.00	11.83	a	0.00	28.86	c	0.00	***
Geranic acid	22.63	c	0.57	15.52	b	0.39	9.82	a	0.25	***	3.90	a	0.10	16.89	b	0.42	28.22	c	0.71	***
Nerolic acid	11.85	b	-	6.87	a	-	12.74	b	-	**	7.86	a	-	7.46	a	-	7.79	a	-	ns
Pentanoic acid	5.60	a	0.03	4.89	a	0.03	4.57	a	0.03	ns	4.33	a	0.03	3.34	a	0.02	7.52	b	0.05	***
Isobutyric acid	71.46	a	0.00	62.27	a	0.00	56.79	a	0.00	ns	59.47	a	0.00	44.18	a	0.00	97.70	b	0.00	***
2-methylbutyric acid	129.05	b	0.04	103.28	a	0.03	100.53	a	0.03	*	101.59	a	0.03	82.43	a	0.03	157.91	b	0.05	***
Hexanoic acid	1694	b	0.56	1172	a	0.39	1284	a	0.43	*	1404	a	0.47	1112	a	0.37	1895	b	0.63	**
Octanoic acid	1583	b	1.58	1005	a	1.01	1188	a	1.19	*	1260	a	1.26	1064	a	1.06	1764	b	1.76	**
Butyric acid	145.76	b	0.07	125.67	ab	0.06	113.69	a	0.05	ns	123.28	a	0.06	95.14	a	0.04	181.42	b	0.08	***
Acetic acid	943.41	a	0.05	836.36	a	0.04	752.64	a	0.04	ns	742.72	b	0.04	532.18	a	0.03	1.223	c	0.06	***
Isovaleric acid	226.82	b	6.87	177.23	a	5.37	172.51	a	5.23	*	183.38	a	5.56	145.08	a	4.40	279.65	b	8.47	***
**γ-Lactones**	710.21	b	20.29	549.35	a	15.70	536.13	a	15.32	*	568.06	a	16.23	448.49	a	12.81	946.18	b	27.03	***
γ-butyrolactone	710.21	b	20.29	549.35	a	15.70	536.13	a	15.32	*	568.06	a	16.23	448.49	a	12.81	946.18	b	27.03	***
**Carbonyl compounds**	263.03	a	0.00	216.61	a	0.00	241.13	a	0.00	ns	260.67	a	0.00	308.83	a	0.00	467.76	b	0.00	***
Acetoin	263.03	a	0.00	216.61	a	0.00	241.13	a	0.00	ns	260.67	a	0.00	308.83	a	0.00	467.76	b	0.00	***
**Others**	33.10	b	0.01	19.74	a	0.01	21.21	a	0.01	***	22.34	b	0.01	18.36	a	0.01	29.45	c	0.01	***
Benzaldehyde	5.70	b	0.00	3.44	a	0.00	3.67	a	0.00	***	2.49	a	0.00	2.07	a	0.00	3.81	b	0.00	***
Hexanal	3.29	b	0.01	1.52	a	0.00	1.04	a	0.00	***	1.50	a	0.00	1.38	a	0.00	2.65	b	0.01	**
Vanillyl acetone	24.11	b	-	14.78	a	-	16.49	a	-	***	18.36	b	-	14.92	a	-	22.99	c	-	***
**Furanic compounds**	2222	a	0.13	1765	a	0.12	1599	a	0.10	ns	1363	ab	0.08	1043	a	0.06	1819	b	0.11	*
Furfural	907.69	a	0.06	818.98	a	0.06	665.66	a	0.05	ns	425.43	a	0.03	344.30	a	0.02	701.77	b	0.05	*
5-methylfurfural	887.43	a	0.04	568.59	a	0.03	644.83	a	0.03	ns	567.19	a	0.03	544.98	a	0.03	650.74	a	0.03	ns
Furfuryl alcohol	427.80	b	0.03	377.29	ab	0.03	288.13	a	0.02	ns	370.22	b	0.02	153.37	a	0.01	466.33	b	0.03	**
**Lactones**	850.69	b	11.80	559.24	a	7.61	767.89	b	9.37	*	601.85	a	8.23	510.70	a	6.60	980.79	b	12.04	***
*cis*-whiskey-lactone	499.21	b	10.85	320.50	a	6.97	383.03	a	8.33	**	346.68	a	7.54	274.00	a	5.96	493.27	b	10.72	**
*trans*-whiskey-lactone	351.48	b	0.95	238.74	a	0.65	384.86	b	1.04	**	255.18	a	0.69	236.71	a	0.64	487.52	b	1.32	***
**Volatile phenols**	290.29	b	20.89	203.17	a	11.65	202.99	a	16.17	*	244.02	b	15.25	165.80	a	11.17	349.89	c	22.11	***
4-vinylphenol	80.43	b	0.45	69.97	ab	0.39	57.31	a	0.32	ns	76.87	b	0.43	52.55	a	0.29	119.22	c	0.66	***
4-vinylguaiacol	76.79	b	1.92	60.47	ab	1.51	49.91	a	1.25	*	73.19	b	1.83	41.13	a	1.03	104.61	c	2.62	***
Eugenol	95.01	b	15.83	47.18	a	7.86	76.91	b	12.82	**	65.40	a	10.90	49.85	a	8.31	96.17	b	16.03	**
Guaiacol	25.31	b	2.66	17.83	a	1.88	16.92	a	1.78	*	19.72	b	2.08	14.52	a	1.53	26.57	c	2.80	***
4-ethylphenol	12.75	c	0.02	7.71	b	0.01	1.94	a	0.00	***	8.84	b	0.01	7.76	b	0.01	3.30	a	0.01	***
**Phenolic aldehydes**	277.41	b	1.39	197.33	a	0.99	200.28	a	1.00	*	200.49	b	1.00	114.35	a	0.57	242.19	b	1.21	***
Vanillin	277.41	b	1.39	197.33	a	0.99	200.28	a	1.00	*	200.49	b	1.00	114.35	a	0.57	242.19	b	1.21	***

The different letters in the same line indicate statistically significant differences. The stars indicate the level of significant differences at 95% (*), 99% (**), and 99.9% (***). ns indicates that no significant differences were found. LT: light toasting; MT: medium toasting; MLT: medium long toasting.

**Table 3:** Concentration (µg 4-nonanol/L) and odour active values (OAVs) of volatile compounds in wines after 12 and 18 months of ageing. MANOVA statistical analysis and effect of grain size on Tempranillo red wines aged in *Quercus petraea* oak barrels.

	12 Months					18 Months				
	SG		EG			SG		EG		
	µg/L	OAV	µg/L	OAV	Sig.	µg/L	OAV	µg/L	OAV	Sig.
**C6 Alcohols**	1613	0.48	1066	0.33	**	1247	0.36	1747	0.50	**
1-hexanol	1498	0.19	1013	0.13	**	1163	0.15	1628	0.20	**
Z-3-hexenol	76.64	0.19	51.94	0.13	**	60.62	0.15	83.45	0.21	*
E-3-hexenol	26.33	0.07	17.78	0.04	***	20.26	0.05	28.96	0.07	*
E-2-hexenol	0.35	0.00	0.95	0.00	***	0.66	0.00	2.64	0.01	***
Z-2-hexenol	12.06	0.03	9.93	0.02	ns	3.60	0.01	3.54	0.01	ns
**Higher alcohols**	124,856	13.75	80,422	10.19	***	98,213	11.62	126,902	14.10	*
1-propanol	438.26	0.00	258.60	0.00	***	388.34	0.00	425.33	0.00	ns
2-octanol	481.81	4.02	468.88	3.91	ns	474.11	3.95	496.35	4.14	*
Isobutanol	2699	0.07	1666	0.04	***	2133	0.05	2576	0.06	ns
1-butanol	127.44	0.00	78.32	0.00	***	108.98	0.00	124.64	0.00	ns
Isoamyl alcohol	39,253	1.31	24,955	0.83	***	30,521	1.02	38,709	1.29	*
3-methyl-1-pentanol	56.90	0.00	38.14	0.00	***	43.45	0.00	59.19	0.00	*
2.3-butanediol	10.17	0.00	5.69	0.00	***	3.24	0.00	8.87	0.00	***
3-methylthiopropanol	206.89	0.21	128.56	0.13	***	165.73	0.17	185.72	0.19	ns
Benzyl alcohol	116.64	0.00	73.35	0.00	***	88.68	0.00	101.77	0.00	ns
2-phenylethanol	81,465	8.15	52,749	5.27	***	64,286	6.43	84,215	8.42	*
**C_13_-norisoprenoids**	4.77	79.93	3.04	52.69	***	3.40	57.87	3.99	67.46	*
α-ionone	0.56	6.20	0.40	4.45	**	0.88	9.82	0.93	10.30	ns
β-ionone	1.18	13.12	0.51	5.69	***	0.27	2.95	0.45	5.02	***
β-damascenone	3.03	60.61	2.13	42.55	*	2.25	45.10	2.61	52.14	ns
**Terpenes**	104.80	4.59	68.61	2.93	***	79.66	3.44	78.35	3.23	ns
Terpinolene	0.40	0.01	0.41	0.01	ns	0.37	0.01	0.76	0.02	***
α-terpineol	5.66	0.01	4.69	0.00	ns	4.32	0.00	5.13	0.01	*
E-geraniol	50.66	2.53	31.62	1.58	***	36.38	1.82	24.77	1.24	***
Z-geraniol (Nerol)	37.74	1.89	23.75	1.19	***	28.76	1.44	35.67	1.78	*
β-citronellol	2.45	0.02	0.54	0.01	***	1.00	0.01	2.74	0.03	***
Linalool	2.97	0.12	2.18	0.09	*	2.87	0.11	3.48	0.14	ns
β-pinene	0.43	0.00	0.29	0.00	***	0.62	0.00	0.72	0.00	*
4-carvomenthenol	4.01	-	3.65	-	ns	4.11	-	4.65	-	ns
Citral	0.47	0.02	1.50	0.05	***	1.23	0.04	0.43	0.02	***
**Ethyl esters and acetates**	72,851	222.56	46,723	156.36	***	56,987	140.24	70,586	199.43	*
Ethyl butyrate	288.68	14.43	202.47	10.12	**	226.66	11.33	313.29	15.66	**
Ethyl 2-methylbutyrate	30.54	1.70	21.13	1.17	**	23.46	1.30	31.84	1.77	*
Ethyl decanoate	37.94	0.19	26.71	0.13	**	15.55	0.08	19.88	0.10	*
Ethyl isovalerate	55.85	18.62	39.20	13.07	**	42.25	14.08	58.96	19.65	*
Ethyl myristate	15.41	0.01	10.05	0.01	***	9.98	0.00	14.23	0.01	*
Methyl salicylate	3.73	37.27	2.44	24.40	***	1.78	17.80	3.00	30.02	**
Hexyl acetate	217.48	0.32	155.48	0.23	**	175.58	0.26	209.90	0.31	ns
Methyl vanillate	8.92	0.00	5.73	0.00	**	6.18	0.00	8.87	0.00	*
Ethyl vanillate	519.11	0.52	317.08	0.32	***	366.46	0.37	471.60	0.48	*
Ethyl hexanoate	608.42	43.46	427.49	30.53	**	445.56	31.83	625.85	44.70	*
Ethyl lactate	44,811	0.29	28,037	0.18	***	35,029	0.23	42,193	0.27	ns
Ethyl octanoate	440.43	88.09	320.26	64.05	**	248.02	49.60	339.48	67.90	*
Diethyl succinate	25,317	4.22	16,816	2.80	**	20,027	3.34	25,771	4.30	*
Isoamyl acetate	390.36	13.01	271.61	9.05	*	290.71	9.69	414.52	13.82	**
2-phenylethyl acetate	106.26	0.43	70.63	0.28	**	79.10	0.32	109.92	0.44	**
**Fatty acids**	4869	9.85	3194	6.38	***	3699	7.69	4767	9.47	**
Propanoic acid	24.48	0.00	13.27	0.00	***	19.35	0.00	21.93	0.00	ns
Geranic acid	21.36	0.53	10.63	0.27	***	12.77	0.32	19.91	0.50	**
Nerolic acid	11.04	-	9.94	-	ns	5.69	-	9.72	-	***
Pentanoic acid	6.38	0.04	3.66	0.02	***	4.93	0.03	5.20	0.03	ns
Isobutyric acid	77.12	0.00	49.89	0.00	***	62.21	0.00	72.03	0.00	ns
2-methylbutyric acid	135.53	0.05	86.38	0.03	***	105.85	0.04	122.10	0.04	ns
Hexanoic acid	1647	0.55	1119	0.37	**	1236	0.41	1705	0.57	**
Octanoic acid	1489	1.49	1029	1.03	**	1125	1.12	1601	1.60	**
Butyric acid	158.36	0.07	98.39	0.04	***	124.53	0.06	142.02	0.06	ns
Acetic acid	1065	0.05	623.10	0.03	***	816.03	0.04	849.53	0.04	ns
Isovaleric acid	233.22	7.07	151.15	4.58	***	186.93	5.66	218.48	6.62	ns
**γ-Lactones**	737.27	21.06	459.85	13.14	***	602.54	17.22	705.95	20.17	ns
γ-butyrolactone	737.27	21.06	459.85	13.14	***	602.54	17.22	705.95	20.17	ns
**Carbonyl compounds**	281.31	0.00	199.20	0.00	***	241.26	0.00	450.25	0.00	***
Acetoin	281.31	0.00	199.20	0.00	***	241.26	0.00	450.25	0.00	***
**Others**	29.78	0.01	19.59	0.01	***	20.24	0.01	26.53	0.01	***
Benzaldehyde	5.30	0.00	3.24	0.00	***	3.03	0.00	2.54	0.00	ns
Hexanal	2.42	0.01	1.48	0.00	**	1.30	0.00	2.39	0.01	**
Vanillyl acetone	22.06	-	14.86	-	***	15.91	-	21.60	-	***
**Furanic compounds**	2932	0.18	792.55	0.10	***	1803	0.11	1013	0.07	***
Furfural	1191	0.08	403.50	0.06	***	588.03	0.04	392.97	0.03	*
5-methylfurfural	1142	0.06	258.30	0.02	***	782.92	0.04	392.36	0.02	***
Furfuryl alcohol	598.06	0.04	130.75	0.02	***	432.14	0.03	227.81	0.02	**
**Lactones**	812.41	9.60	639.47	9.58	*	584.45	6.36	811.11	11.55	**
*cis*-whiskey-lactone	389.19	8.46	412.64	8.97	ns	251.25	5.46	491.38	10.68	***
*trans*-whiskey-lactone	423.23	1.14	226.82	0.61	***	333.21	0.90	319.73	0.86	ns
**Volatile phenols**	288.21	20.85	176.09	11.62	***	243.63	16.24	262.84	16.11	ns
4-vinylphenol	85.09	0.47	53.38	0.30	**	73.76	0.41	92.00	0.51	*
4-vinylguaiacol	71.90	1.80	52.88	1.32	*	70.99	1.77	74.96	1.87	ns
Eugenol	93.00	15.50	53.07	8.85	***	69.85	11.64	71.11	11.85	ns
Guaiacol	29.16	3.07	10.89	1.15	***	22.84	2.40	17.69	1.86	**
4-ethylphenol	9.06	0.01	5.87	0.01	**	6.19	0.01	7.08	0.01	ns
**Phenolic aldehydes**	330.15	1.65	119.86	0.60	***	216.48	1.08	154.87	0.77	**
Vanillin	330.15	1.65	119.86	0.60	***	216.48	1.08	154.87	0.77	**

The stars indicate the level of significant differences at 95% (*), 99% (**), and 99.9% (***). ns indicates that no significant differences were found. SG: standard grain size; EG: extra fine grain size.

**Table 4:** Combined effect of toasting level and grain size after 12 months of ageing in *Quercus petraea* oak barrels. Concentration (µg 4-nonanol/L) of volatile compounds in wines.

	LT × SG		LT × EG		MT × SG		MT × EG		MLT × SG		MLT × EG		Sig.
**C6 Alcohols**	2325	b	1073	a	1199	a	1040	a	1316	a	1083	a	***
1-hexanol	2148	b	983.53	a	1117	a	969.35	a	1229	a	1087	a	**
Z-3-hexenol	108.86	b	49.85	a	58.35	a	49.79	a	62.70	a	56.19	a	***
E-3-hexenol	37.67	b	16.87	a	20.03	a	16.17	a	21.28	a	20.29	a	***
E-2-hexenol	0.47	b	0.16	a	0.18	a	1.07	c	0.40	b	1.64	d	***
Z-2-hexenol	29.63	c	22.65	b	3.29	a	3.90	a	3.26	a	3.24	a	***
**Higher alcohols**	172,903	b	75,902	a	99,426	a	78,993	a	102,238	a	86,372	a	***
1-propanol	557.22	c	232.82	a	407.42	b	276.86	a	350.14	ab	266.30	a	**
2-octanol	504.34	b	471.88	a	464.83	a	464.80	a	476.28	a	469.96	a	*
Isobutanol	3485	c	1498	a	2524	b	1791.32	a	2090	ab	1709	a	***
1-butanol	166.18	c	71.97	a	117.01	b	82.81	ab	99.12	ab	80.20	ab	**
Isoamyl alcohol	52,613	b	24,109	a	33,434	a	24,512	a	31,713	a	26,244	a	***
3-methyl-1-pentanol	82.77	b	36.51	a	41.69	a	36.78	a	46.24	a	41.12	a	***
2.3-butanediol	15.02	d	4.80	a	8.32	c	6.85	a	7.19	b	5.43	a	***
3-methylthiopropanol	274.70	b	129.99	a	185.24	a	122.52	a	160.72	a	133.18	a	**
Benzyl alcohol	160.55	b	73.62	a	100.16	a	67.96	a	89.19	a	78.47	a	***
2-phenylethanol	115,045	b	49,273	a	62,143	a	51,631	a	67,207	a	57,344	a	***
**C_13_-norisoprenoids**	7.64	b	3.24	a	3.38	a	2.65	a	3.30	a	3.23	a	***
α-ionone	0.45	bc	0.31	ab	0.76	d	0.20	ab	0.47	c	0.69	d	***
β-ionone	2.71	b	0.67	a	0.46	a	0.44	a	0.37	a	0.43	a	***
β-damascenone	4.48	b	2.26	a	2.15	a	2.01	a	2.46	a	2.11	a	**
**Terpenes**	138.00	c	64.20	a	94.68	b	64.70	a	81.71	ab	76.93	ab	***
Terpinolene	0.00	a	0.79	d	0.77	d	0.24	a	0.43	c	0.19	b	***
α-terpineol	8.18	b	5.07	a	4.52	a	4.16	a	4.29	a	4.84	a	*
E-geraniol	67.26	b	29.84	a	41.74	a	28.56	a	42.98	a	36.45	a	***
Z-geraniol (Nerol)	47.86	c	22.66	a	36.54	b	23.57	a	28.83	ab	25.04	a	***
β-citronellol	4.27	e	0.24	a	1.00	c	0.53	a	2.09	d	0.84	bc	***
Linalool	4.36	c	1.58	a	2.76	b	2.83	a	1.77	ab	2.13	ab	***
β-pinene	0.26	b	0.13	a	0.26	b	0.32	a	0.77	d	0.42	c	***
4-carvomenthenol	5.52	d	3.11	b	6.22	d	4.28	a	0.30	a	3.55	bc	***
Citral	0.29	a	0.80	b	0.86	b	0.22	a	0.26	a	3.47	c	***
**Ethyl esters and acetates**	102,561	b	45,474	a	58,409	a	45,525	a	57,582	a	49,170	a	***
Ethyl butyrate	421.71	b	189.51	a	203.16	a	193.92	a	241.17	a	223.98	a	**
Ethyl 2-methylbutyrate	47.29	b	20.40	a	20.63	a	20.11	a	23.71	a	22.86	a	**
Ethyl decanoate	45.05	c	19.90	a	36.65	bc	34.17	a	32.12	abc	26.05	ab	*
Ethyl isovalerate	88.71	b	38.18	a	35.03	a	37.33	a	43.82	a	42.09	a	***
Ethyl myristate	21.62	c	9.16	a	13.21	b	10.25	ab	11.39	ab	10.74	ab	***
Methyl salicylate	6.99	b	1.85	a	2.20	a	2.98	a	1.99	a	2.49	a	***
Hexyl acetate	378.80	c	175.56	b	99.05	a	161.23	b	174.59	b	129.65	ab	***
Methyl vanillate	12.77	b	4.75	a	7.01	a	5.98	a	6.98	a	6.47	a	***
Ethyl vanillate	773.02	b	288.66	a	350.85	a	312.66	a	433.46	a	349.92	a	***
Ethyl hexanoate	929.03	b	425.74	a	422.31	a	412.58	a	473.92	a	444.15	a	**
Ethyl lactate	59,906	b	27,091	a	39,904	a	27,613	a	34,622	a	29,407	a	***
Ethyl octanoate	646.61	b	291.34	a	346.72	a	384.56	a	327.95	a	284.88	a	***
Diethyl succinate	38,554	b	16,601	a	16,620	a	16,001	a	20,778	a	17,845	a	***
Isoamyl acetate	572.02	b	250.67	a	273.58	a	267.06	a	325.49	a	297.09	a	**
2-phenylethyl acetate	157.41	b	66.45	a	74.82	a	68.52	a	86.54	a	76.92	a	**
**Fatty acids**	6774	b	2938	a	3898	a	3157	a	3936	a	3487	a	***
Propanoic acid	31.04	d	11.95	a	23.71	c	13.96	a	18.70	bc	13.90	ab	***
Geranic acid	31.88	c	13.38	b	13.69	b	17.35	b	18.50	b	1.14	a	***
Nerolic acid	17.98	d	5.72	ab	4.58	a	9.16	c	10.55	c	14.92	d	***
Pentanoic acid	8.11	c	3.09	a	5.62	b	4.17	a	5.42	b	3.71	ab	***
Isobutyric acid	95.23	c	47.69	a	74.18	bc	50.36	ab	61.95	ab	51.62	ab	**
2-methylbutyric acid	177.35	c	80.75	a	120.85	b	85.70	a	108.37	ab	92.68	ab	***
Hexanoic acid	2339	b	1050	a	1261	a	1082	a	1342	a	1225	a	***
Octanoic acid	2218	b	948.38	a	1011	a	999.10	a	1238	a	1138	a	**
Butyric acid	201.37	c	90.15	a	152.46	b	98.88	a	121.24	ab	106.14	a	**
Acetic acid	1343	d	543.59	a	1025	c	648.01	a	827.58	bc	677.70	ab	***
Isovaleric acid	311.17	b	142.47	a	205.70	a	148.76	a	182.79	a	162.22	a	***
**γ-Lactones**	987.77	c	432.64	a	648.40	b	450.29	ab	575.64	ab	496.62	ab	***
γ-butyrolactone	987.77	c	432.64	a	648.40	b	450.29	ab	575.64	ab	496.62	ab	***
**Carbonyl compounds**	340.59	c	185.46	a	239.04	ab	194.18	a	264.31	b	217.95	ab	**
Acetoin	340.59	c	185.46	a	239.04	ab	194.18	a	264.31	b	217.95	ab	**
**Others**	46.56	b	19.63	a	20.70	a	18.79	a	22.07	a	20.34	a	***
Benzaldehyde	7.45	d	3.95	bc	3.74	abc	3.14	a	4.70	c	2.65	a	***
Hexanal	4.42	c	2.15	b	1.78	ab	1.25	a	1.06	a	1.03	a	***
Vanillyl acetone	34.69	b	13.53	a	15.18	a	14.39	a	16.32	a	16.67	a	***
**Furanic compounds**	3678	c	767.84	a	2745	bc	784.88	a	2372	b	824.92	a	***
Furfural	1413	c	402.69	a	1279	c	358.37	a	881.88	b	449.43	a	***
5-methylfurfural	1539	c	235.81	a	875.82	ab	261.36	a	1012	bc	277.74	a	**
Furfuryl alcohol	726.25	c	129.34	a	589.43	bc	165.16	a	478.50	b	97.75	a	***
**Lactones**	1124	c	577.23	b	470.71	a	647.77	ab	842.38	b	693.39	b	**
*cis*-whiskey-lactone	648.37	c	350.05	b	170.75	a	470.25	b	348.44	b	417.62	b	***
*trans*-whiskey-lactone	475.78	b	227.18	a	299.96	a	177.52	a	493.94	b	275.77	a	***
**Volatile phenols**	402.40	b	178.17	a	232.24	a	174.10	a	229.98	a	176.00	a	***
4-vinylphenol	107.50	c	53.36	a	86.09	bc	53.84	a	61.67	ab	52.95	a	**
4-vinylguaiacol	97.21	b	56.37	a	67.54	a	53.41	a	50.95	a	48.87	a	*
Eugenol	141.46	c	48.55	a	44.95	a	49.42	a	92.59	b	61.23	a	***
Guaiacol	39.37	c	11.26	a	25.42	b	10.24	a	22.69	b	11.16	a	***
4-ethylphenol	16.85	c	8.64	b	8.24	b	7.19	a	2.09	a	1.79	a	***
**Phenolic aldehydes**	459.12	c	95.71	a	272.76	b	121.89	a	258.58	b	141.98	a	***
Vanillin	459.12	c	95.71	a	272.76	b	121.89	a	258.58	b	141.98	a	***

The different letters in the same line indicate statistically significant differences. The stars indicate the level of significant differences at 95% (*), 99% (**), and 99.9% (***). ns indicates that no significant differences were found. LT: light toasting; MT: medium toasting; MLT: medium long toasting; SG: standard grain; EG: extra fine grain.

**Table 5:** Combined effect of toasting level and grain size after 18 months of ageing in *Quercus petraea* oak barrels. Concentration (µg 4-nonanol/L) of volatile compounds in wines.

	LT × SG		LT × EG		MT × SG		MT × EG		MLT × SG		MLT × EG		Sig.
**C6 Alcohols**	1395	a	1433	a	1055	a	1116	a	1293	a	2691	b	***
1-hexanol	1302	a	1338	a	984.71	a	1041	a	1201	a	2506	b	***
Z-3-hexenol	66.72	a	68.51	a	50.32	a	53.51	a	64.83	a	128.32	b	***
E-3-hexenol	22.79	a	23.06	a	16.40	a	17.96	a	21.58	a	45.88	b	***
E-2-hexenol	0.85	a	1.95	b	0.37	a	0.70	a	0.76	a	5.27	c	***
Z-2-hexenol	2.93	b	1.57	a	2.89	b	3.14	b	4.96	c	5.91	c	***
**Higher alcohols**	104,843	a	101,534	a	78,751	a	81,091	a	111,045	a	198,080	b	***
1-propanol	396.44	ab	304.58	a	258.35	a	267.24	a	510.22	b	704.16	c	***
2-octanol	485.83	a	492.31	a	465.56	a	471.29	a	470.93	a	525.45	b	*
Isobutanol	2499	a	1882	a	1599	a	1621	a	2302	a	4226	b	***
1-butanol	119.68	bc	88.34	ab	77.82	a	81.31	ab	129.43	c	204.28	d	***
Isoamyl alcohol	34,429	a	28,306	a	24,177	a	24,578	a	32,957	a	63,243	b	***
3-methyl-1-pentanol	49.00	a	49.66	a	35.54	a	38.94	a	45.81	a	88.97	b	**
2.3-butanediol	2.39	a	9.13	c	0.83	a	4.85	b	6.49	b	12.61	d	***
3-methylthiopropanol	176.22	bc	136.68	ab	116.91	a	110.23	a	204.07	c	310.25	d	***
Benzyl alcohol	94.35	ab	76.35	ab	68.92	a	70.24	a	102.78	b	158.71	c	***
2-phenylethanol	66,591	a	70,189	a	51,952	a	53,848	a	74,316	a	128,607	b	***
**C_13_-norisoprenoids**	2.85	a	3.54	a	2.66	a	3.50	a	4.70	b	4.92	b	***
α-ionone	0.59	a	0.91	b	0.73	ab	0.64	a	1.33	c	1.23	c	***
β-ionone	0.32	c	0.40	d	0.18	a	0.72	e	0.30	bc	0.23	ab	***
β-damascenone	1.94	a	2.23	a	1.75	a	2.14	a	3.08	b	3.46	b	**
**Terpenes**	87.24	b	74.03	ab	67.78	a	73.52	ab	83.96	ab	87.49	b	ns
Terpinolene	0.54	c	1.13	e	0.12	a	0.67	d	0.45	b	0.47	b	***
α-terpineol	4.99	c	4.39	abc	3.38	a	3.79	ab	4.58	bc	7.22	d	***
E-geraniol	41.48	b	35.95	b	32.60	b	33.71	b	35.08	b	4.64	a	***
Z-geraniol (Nerol)	30.42	a	23.93	a	24.59	a	26.65	a	31.27	a	56.42	b	***
β-citronellol	0.34	a	2.22	d	1.59	c	1.96	cd	1.06	b	4.04	e	***
Linalool	2.88	a	2.09	a	2.15	a	2.47	a	3.57	a	5.88	b	***
β-pinene	1.08	d	0.53	c	0.47	bc	0.36	ab	0.30	a	1.26	e	***
4-carvomenthenol	3.56	a	3.53	a	2.54	a	3.59	a	6.23	b	6.84	b	***
Citral	1.93	d	0.24	a	0.34	a	0.33	a	1.42	c	0.72	b	***
**Ethyl esters and acetates**	62,428	a	54,064	a	45,421	a	46,342	a	63,111	a	111,351	b	***
Ethyl butyrate	245.52	a	261.42	a	202.25	a	205.63	a	232.22	a	472.81	b	***
Ethyl 2-methylbutyrate	25.95	a	26.64	a	19.53	a	19.85	a	24.90	a	49.04	b	***
Ethyl decanoate	13.92	ab	12.69	a	11.88	a	9.73	a	20.85	b	37.23	c	***
Ethyl isovalerate	47.14	a	49.86	a	36.33	a	37.24	a	43.30	a	89.77	b	**
Ethyl myristate	9.66	a	8.75	a	9.58	a	10.28	a	10.71	a	23.67	b	***
Methyl salicylate	1.07	a	0.48	a	1.65	ab	2.92	b	2.62	b	5.61	c	***
Hexyl acetate	171.46	b	205.62	b	151.41	b	35.80	a	203.89	b	388.27	c	***
Methyl vanillate	7.12	a	7.23	a	5.21	a	5.85	a	6.20	a	13.52	b	**
Ethyl vanillate	396.37	a	405.50	a	329.98	a	318.08	a	373.02	a	691.21	b	***
Ethyl hexanoate	504.43	a	506.35	a	371.95	a	375.38	a	460.31	a	995.83	b	***
Ethyl lactate	40,519	a	30,832	a	26,989	a	27,348	a	37,578	a	68,399	b	***
Ethyl octanoate	275.23	a	266.31	a	188.54	a	128.16	a	280.30	a	623.97	b	***
Diethyl succinate	19,821	a	21,061	a	16,777	a	17,496	a	23,482	a	38,757	b	***
Isoamyl acetate	309.61	a	333.60	a	254.53	a	275.33	a	308.00	a	634.63	b	**
2-phenylethyl acetate	81.04	a	86.40	a	72.00	a	74.49	a	84.27	a	168.88	b	***
**Fatty acids**	3960	a	3865	a	3163	a	3066	a	3973	a	7370	b	***
Propanoic acid	21.33	b	21.13	b	14.10	ab	9.57	a	22.63	b	35.09	c	***
Geranic acid	5.65	a	2.15	a	16.47	b	17.31	b	16.18	b	40.27	c	***
Nerolic acid	5.44	a	10.28	b	6.67	a	8.25	ab	4.95	a	10.62	b	**
Pentanoic acid	4.41	a	4.25	a	3.50	a	3.18	a	6.89	b	8.16	b	***
Isobutyric acid	66.95	ab	51.99	ab	46.56	ab	41.80	a	73.12	b	122.29	c	***
2-methylbutyric acid	114.24	ab	88.93	ab	81.97	a	82.89	a	121.33	b	194.49	c	***
Hexanoic acid	1381	a	1427	a	1085	a	1139	a	1240	a	2549	b	***
Octanoic acid	1195	a	1325	a	1052	a	1076	a	1127	a	2401	b	***
Butyric acid	142.52	a	104.05	a	95.44	a	94.83	a	135.63	a	227.20	b	***
Acetic acid	817.44	bc	668.00	ab	615.90	ab	448.45	a	1014	c	1432	d	***
Isovaleric acid	205.44	a	161.31	a	145.03	a	145.13	a	210.32	a	348.98	b	***
**γ-Lactones**	631.75	ab	504.38	ab	446.87	a	450.12	a	728.99	b	1163	c	***
γ-butyrolactone	631.75	ab	504.38	ab	446.87	a	450.12	a	728.99	b	1163	c	***
**Carbonyl compounds**	307.54	b	213.80	ab	158.74	a	458.91	c	257.50	ab	678.02	d	***
Acetoin	307.54	b	213.80	ab	158.74	a	458.91	c	257.50	ab	678.02	d	***
**Others**	21.63	ab	23.05	b	19.54	ab	17.19	a	19.55	ab	39.35	c	***
Benzaldehyde	2.96	bc	2.02	ab	2.36	ab	1.78	a	3.77	c	3.84	c	***
Hexanal	1.12	a	1.87	a	1.63	a	1.12	a	1.13	a	4.17	b	***
Vanillyl acetone	17.54	ab	19.17	b	15.54	ab	14.29	a	14.65	ab	31.34	c	***
**Furanic compounds**	1691	bc	1035	b	1761	c	324.13	a	1957	c	1681	bc	**
Furfural	447.50	bc	403.37	b	688.60	bc	0.00	a	628.00	bc	775.54	c	**
5-methylfurfural	751.47	c	382.91	ab	816.02	c	273.93	a	781.25	c	520.23	b	***
Furfuryl alcohol	492.22	c	248.21	b	256.54	b	50.20	a	547.65	c	385.01	bc	**
**Lactones**	503.59	a	700.12	ab	487.42	a	533.99	ab	762.36	b	1199	c	***
*cis*-whiskey-lactone	303.45	ab	389.90	b	164.70	a	383.30	b	285.59	ab	700.95	c	***
*trans*-whiskey-lactone	200.14	a	310.22	b	322.72	b	150.69	a	476.77	c	498.28	c	***
**Volatile phenols**	220.55	bc	267.49	cd	188.13	ab	143.47	a	322.21	de	377.56	e	***
4-vinylphenol	63.28	ab	90.46	bc	55.19	a	49.90	a	102.80	c	135.65	d	***
4-vinylguaiacol	62.50	bc	83.88	cd	50.06	ab	32.20	a	100.42	d	108.80	d	***
Eugenol	64.85	ab	65.96	ab	56.74	a	42.97	a	87.96	bc	104.39	c	**
Guaiacol	21.97	bc	17.46	b	18.39	b	10.64	a	28.17	c	24.97	c	***
4-ethylphenol	7.95	b	9.73	c	7.75	b	7.76	b	2.86	a	3.75	a	***
**Phenolic aldehydes**	244.40	cd	156.58	b	196.97	bc	31.73	a	208.08	bc	276.30	d	***
Vanillin	244.40	cd	156.58	b	196.97	bc	31.73	a	208.08	bc	276.30	d	***

The different letters in the same line indicate statistically significant differences. The stars indicate the level of significant differences at 95% (*), 99% (**), and 99.9% (***). ns indicates that no significant differences were found. LT—Light toasting. MT—medium toasting. MLT—medium long toasting. SG—standard grain. EG—extra fine grain.

**Table 6:** Experimental design: distribution of 36 barrels by toasting level and grain size.

Number of Barrels	Toasting Level ^1^	Grain Size ^1^	Toasting Level × Grain Size
6	LT	SG	LT × SG
6	LT	EG	LT × EG
6	MT	SG	MT × SG
6	MT	EG	MT × EG
6	MLT	SG	MLT × SG
6	MLT	EG	MLT × EG

^1^ LT: light toasting; MT: medium toasting; MLT: medium long toasting; SG: standard grain; EG: extra fine Grain.

**Table 7:** Odour thresholds and aromatic descriptors for analysed volatiles.

Volatile Families and Compounds	Odour Threshold (µg/L)	Descriptor	Ref.
**C6 Alcohols (Non-oak)**			
1-hexanol	8000	Green, grass	[46]
Z-3-hexenol	400	Green, grass, bitter	[46]
E-3-hexenol	400	Green, floral	[47]
E-2-hexenol	400	Green grass, herb	[48]
Z-2-hexenol	400	Green grass, herb	[48]
**Higher alcohols (Non-oak)**			
1-propanol	306,000	Fresh, alcohol	[49]
2-octanol	120	-	[50]
Isobutanol	40,000	Alcohol, solvent, bitter	[46]
1-butanol	150,000	Medicinal, phenolic	[51]
Isoamyl alcohol	30,000	Whiskey, solvent, sweet	[46]
3-methyl-1-pentanol	50,000	Herbaceous, cocoa	[51]
2,3-butanediol	150,000	Fruity	[51]
3-methylthiopropanol	1000	Cabbage, cooked potato	[46]
Benzyl alcohol	200,000	Sweet, fruity	[51]
2-phenylethanol	10,000	Floral, roses, perfume	[50]
**C_13_-norisoprenoids (Non-oak)**			
α-ionone	0.09	Raspberry, violet, sweet fruity	[48]
β-ionone	0.09	Raspberry, violet, sweet fruity	[48]
β-damascenone	0.05	Bark, canned peach, baked apple, dry plum	[48]
**Terpenes (Non-oak)**			
Terpinolene	41	Pine, citrus, earthy	[52]
α-terpineol	1000	Lilac, floral, sweet	[49]
E-geraniol	20	Floral, geranium, rose	[53]
Z-geraniol (Nerol)	20	Floral, rose, lime	[53]
β-citronellol	100	Green lemon	[50]
Linalool	25.2	Fruity, citric	[54]
β-pinene	1500	Pine, hay, green	[52]
4-carvomenthenol	-	Nutmeg	-
Citral	28	Lemon	-
**Ethyl esters and acetates (Non-oak)**			
Ethyl butyrate	20	Papaya, apple	[55]
Ethyl 2-methylbutyrate	18	Fruity, strawberry, anise	[26]
Ethyl decanoate	200	Fruity, fatty, pleasant	[50]
Ethyl isovalerate	3	Banana, sweet fruity	[48]
Ethyl myristate	2000	Sweet fruity, butter, fatty odour	[48]
Methyl salicylate	0.1	Peppermint	[53]
Hexyl acetate	670	Fruity, herbs, apple	[53]
Methyl vanillate	3000	Sweet, vanilla-like	[26]
Ethyl vanillate	990	Sweet, vanilla-like	[26]
Ethyl hexanoate	14	Apple, fruity, sweetish	[51]
Ethyl lactate	154,000	Strawberry, raspberry	[51]
Ethyl octanoate	5	Apple, fruity, sweetish	[51]
Diethyl succinate	6000	Light fruity, wine	[51]
Isoamyl acetate	30	Banana	[51]
2-phenylethyl acetate	250	Floral	[51]
**Fatty acids (Non-oak)**			
Propanoic acid	8100	Vinegarish	[54]
Geranic acid	40	Green	[56]
Nerolic acid	-	Honey, floral	-
Pentanoic acid	160	Black wallnut	-
Isobutyric acid	200,000	Fatty	[50]
2-methylbutyric acid	3000	Buttery, cheesy	[50]
Hexanoic acid	3000	Cheese, fatty	[57]
Octanoic acid	1000	Cheese, fatty, rancid	[51]
Butyric acid	2200	Rancid, cheese, sweat	[58]
Acetic acid	20,000	Vinegar	[51]
Isovaleric acid	33	Acid, rancid	[46]
**γ-Lactones (Non-oak)**			
γ-butyrolactone	35	Caramel, sweet, fruity	[58]
**Carbonyl compounds (Non-oak)**			
Acetoin	150,000	Buttery, cream	[59]
**Others (Non-oak)**			
Benzaldehyde	2000	Bitter almond	[49]
Hexanal	350	Fatty, herbaceous, and green	[59]
Vanillylacetone	-	Ginger	-
**Furanic compounds (oak)**			
Furfural	14,100	Burned almonds, incense	[46,50]
5-methylfurfural	20,000	Bitter almond, spice	[46]
Furfuryl alcohol	15,000	Hay	[46]
**Lactones (oak)**			
*cis*-whiskey-lactone	46	Woody, coconut, vanilla	[50]
*trans*-whiskey-lactone	370	Woody, coconut, vanilla	[50]
**Volatile phenols (oak)**			
4-vinylphenol	180	Medicine, phenolic, paint	[26]
4-vinylguaiacol	40	Spices, clove, curry	[26]
Eugenol	6	Clove, honey, spicy	[50]
Guaiacol	9.5	Smoke, toasted, spicy	[51]
4-ethylphenol	620	Leather, animal	[51]
**Phenolic aldehydes (oak)**			
Vanillin	200	Vanilla	[50]

## Data Availability

The original contributions presented in this study are included in the article. Further inquiries can be directed to the corresponding author.
